# The Swedish longitudinal Gothenland Millennium Cohort for studying wellbeing from early adolescence through adulthood

**DOI:** 10.1136/bmjopen-2025-100327

**Published:** 2025-12-10

**Authors:** Tina M Olsson, Torbjörn Kalin, Sabina Kapetanovic, Russell Turner, Arne Gerdner

**Affiliations:** 1 Department of Social Work, Jönköping University, Jönköping, Sweden; 2 Department of Social Work, University of Gothenburg, Gothenburg, Västra Götaland County, Sweden; 3 Department of Social and Behavioural Studies, University West, Trollhattan, Sweden

**Keywords:** Adolescents, Child protection, Substance misuse, MENTAL HEALTH

## Abstract

**Abstract:**

**Purpose:**

The purpose of this article is to present the Gothenland Millennium Cohort, describe the data collection process, present key measures used and summarise some of the key findings to date in order to stimulate collaboration and use of the cohort data. This research programme was originally established to study pathways to alcohol and drug use, behavioural problems, mental health issues, and the factors that promote or prevent these outcomes. The Cohort aims to support scientific research and doctoral education through a longitudinal study that tracks individuals from early adolescence through adulthood. This programme is multidisciplinary (social work, psychology, disability research) with the goal of producing high-quality research that deepens our understanding of how early-life vulnerabilities, risks and protective factors influence long-term wellbeing, including health and welfare, in diverse populations.

**Participants:**

In 2013, all school-registered adolescents, in grades 6 and 7 (aged 12 and 13), in four municipalities in Gothenland region (ie, southern Sweden) born in year 2000 or 2001, were invited to participate. Of 2150 invited adolescents, 1885 (88%) accepted participation in the programme and 1760 (93.4%) participated in at least one of the annual data collection waves up to grade 9 (Wave 4), with participation rates ranging from 70% to 85% per wave. Wave 5 questionnaires were collected during the second year of upper secondary school (grade 11). In Wave 5, half (50.4 %; *n* = 949) of the adolescents participated. In Wave 6, interviews were conducted with a selection of participants in their last year of upper secondary school (grade 12). Parents were surveyed in Waves 1 and 2 by self-report questionnaires (response rate = 32%; 41%). Data were also gathered from teachers (attrition <30% across Waves 1–4). Child welfare service records and population-based register data have also been collected.

**Findings to date:**

Over 240 publications have been produced as of September 2025 in the areas of disability and everyday functioning, child-parent relationships, child welfare, substance use and criminal behaviour, mental health, trauma, harassment, and sexuality.

**Future plans:**

These include continued investigation of wellbeing and its related indicators during adolescence as well as in emerging adulthood, continued efforts to secure funding and an age 25 expansion of the cohort data.

**STRENGTHS AND LIMITATIONS OF THIS STUDY**

- A strength of the Gothenland Millennium Cohort is its prospective longitudinal design which is representative of a general adolescent population and, due to the size of the cohort, subgroup analyses are possible.
- Additionally, the Gothenland Millennium Cohort includes a wide number of measures across multiple areas of vulnerability, risk, protection, and wellbeing.
- Gothenland Millennium Cohort data include multiple informants as well as population-based register data which allows for investigation from multiple perspectives.
- As the cohort followed is from a general population, certain specific subgroups of adolescents may be small or not fully represented in the cohort.
- Not all measures used are included in every data collection wave, meaning that longitudinal analyses need careful planning in the cohort data.

## Introduction

 Adolescence represents a pivotal phase marked by profound biological,^1^ emotional,^2^ behavioural^3^ and social changes.^4^ This transitional period is the bridge between childhood and adulthood, as individuals adapt to physical transformations and evolving sexual identity, establish personal values and prepare for societal roles through, for example, education and vocational training. Despite the general physical health of most adolescents, many struggle with feelings of disorientation and insecurity. The quest for acceptance within peer groups can sometimes lead to detrimental behaviours, which impact both themselves and others. Emotional distress, substance misuse and antisocial behaviours often find their roots in adolescence, forming a complex interplay of challenges.^5^ While these issues are widely acknowledged, our understanding of their causal relationships remains incomplete. In this article, we present the Gothenland Millennium Cohort, which is a resource for conducting research that can advance our understanding of these relationships. The purpose of this article is to stimulate collaboration and use of Millennium Cohort data by describing the data collection process, key measures used and summarising some of the key findings to date.

The United Nations’^6^ 2030 Agenda for Sustainable Development includes aims to ensure healthy lives and promote well-being for all ages (Sustainable Development Goal 3). Well-being is influenced by social, economic and environmental conditions and encompasses quality of life and the ability to contribute to society with a sense of meaning and purpose.^7^ It has subjective (eg, how an individual experiences their life) and objective (eg, compares life circumstances with social norms and values) components.^8^ Increasing our understanding of well-being, its component parts, vulnerabilities, along with risks and protective factors in diverse groups of people supports the tracking of the equitable distribution of health and welfare in society. Well-being is a key component of health.^9^ Indicators of well-being include quality of life measures (eg, health status, education, social connections, personal security and subjective well-being) and material living conditions (eg, income and wealth, employment and earnings and housing^10^). Many young people, however, report poor well-being.^11 12^ A decline in well-being has occurred among both young males and females aged 16–24 since 2000.^13^,^15^ Sustained deficits in well-being during adolescence could pose a risk for poor health and mental health issues in adulthood.^9 16^

Research investigating the mental health needs of young people aged 12–24 years has found a strong relationship between reduced well-being and many other health and development concerns (eg, educational achievement, substance use and misuse, and violence^13^). Most threats to well-being emerge in this age, although they are not detected until later in life (ibid.). Evidence is available in support of a multifactorial cause for deficits in well-being in young people. Poverty and social disadvantage are strongly associated with poor well-being.^14 15 17^ This association is complex and bidirectional.^13^ Young people in families with parental mental disorder, substance misuse or parental discord are at greater risk of displaying deficits in well-being.^18^,^20^ Violence, child abuse, historical disadvantage and cultural factors are also risk factors^21^ for later deficiencies in well-being.

The Gothenland Millennium Cohort endeavours to provide a platform to cultivate scientific discovery and foster doctoral education focused on a comprehensive longitudinal study spanning from early adolescence into adulthood and beyond. This initiative is a multidisciplinary and collaborative enterprise uniting the disciplines of social work, psychology and disability research and fortified by a common, multifactorial dataset across various research domains. Born from the research project Longitudinal Research on Development In Adolescence (LoRDIA) and its successor Longitudinal Research on Development to Young Adults (LoRDYA), the programme’s overarching aim was initially to study various paths into alcohol and drug use and misuse, behavioural problems, mental health issues and factors that promote or prevent these trajectories. This has expanded over the programme’s life as the data collected has proved rich in explanatory power. Long-term longitudinal data are imperative to the conduct of high-quality research which can increase our understanding of how vulnerability, risk and protection early in life among diverse populations impact well-being (eg, health, welfare) into adulthood. The Gothenland Millennium Cohort provides a robust empirical foundation for this type of research. At the time of writing, the adolescents in the cohort—all born in year 2000 or 2001—have been followed from age 12 or 13, and the most recent data collection was conducted when participants were 20 or 21 years old.

## Cohort description

### Study population and recruitment

The Gothenland Millennium Cohort is a total population prospective sample based on four small and medium-sized municipalities (<40 000 inhabitants) located in Gothenland, Sweden (Swedish: *Götaland*). Two of the municipalities included were industrial and two were characterised as commuter towns to nearby cities. One of the commuter municipalities was near a large city. The unemployment rate, annual income, educational level and proportion of first-generation immigrants across the four municipalities was similar to national averages.^22^

**Figure 1:**
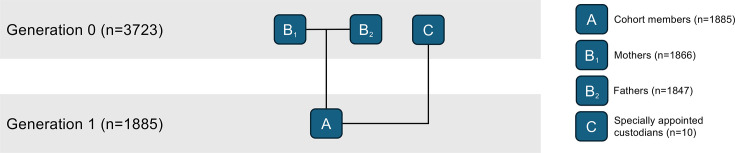
Overview of generations within the cohort.

**Figure 2:**
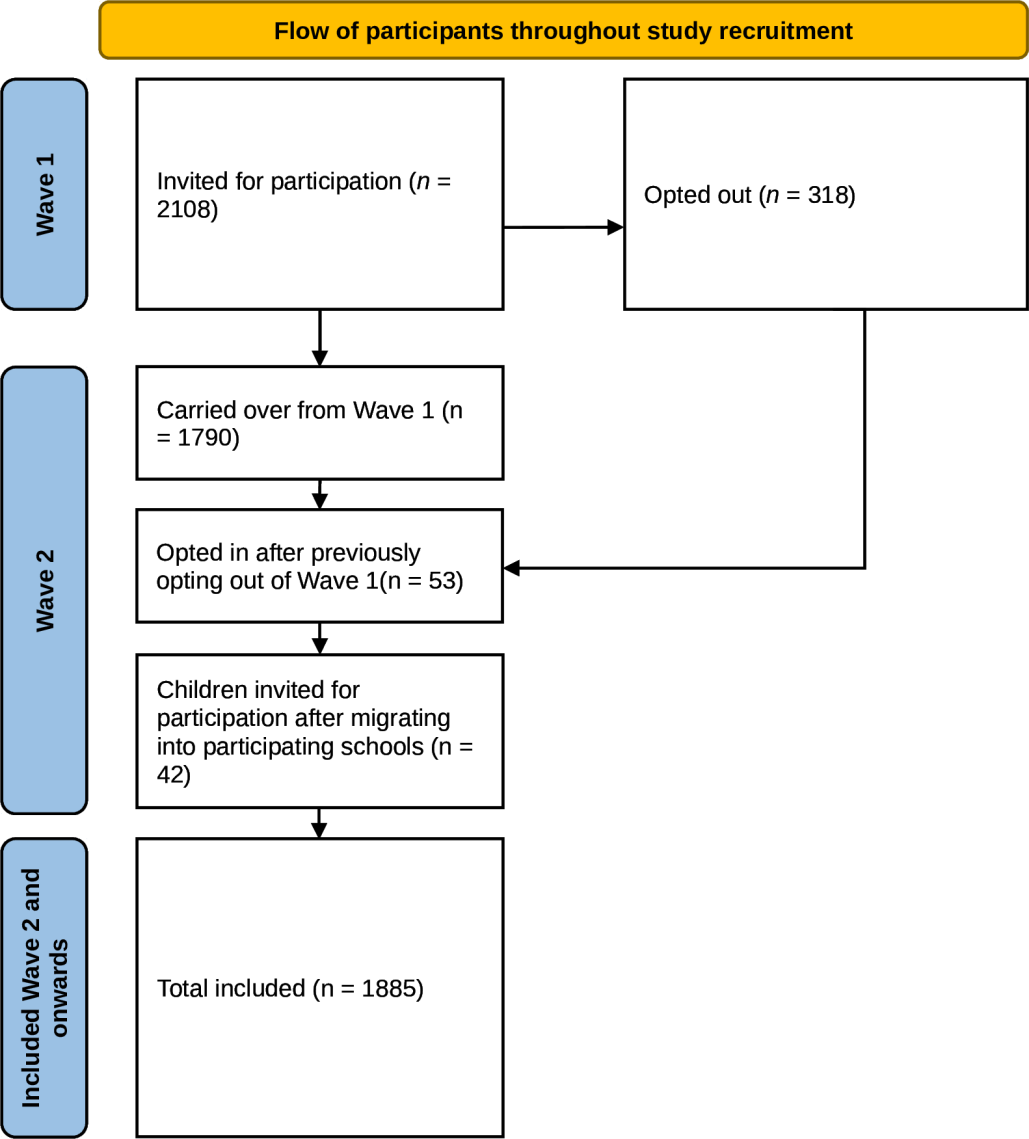
Flow of participants through study recruitment.

Basic demographic information on the cohort can be found in table 1. All adolescents (n=2108) who were registered in grades 6 and 7, born in the millennium years 2000 or 2001, were invited to take part in the research programme in 2013 when they were 12 or 13 years old. Thus, the cohort comprised two whole school years or grades. In 2014, 42 new adolescents who had recently migrated into participating communities were also invited (figure 1). The total invited population was therefore 2150. As the participants were minors, parents/guardians (hereafter: parents) were provided information about the aims and scope of the programme, its longitudinal character, the voluntary basis and their right to decline participation on behalf of their child via regular mail, e-mail or telephone. The information was translated and provided in 33 languages with the intent to reach all possible participants and their parents in their mother tongue. The letter was sent to each parent if they lived separately. Information provided during recruitment directly to adolescents was adapted for their age. Adolescents were informed of their right to decide for themselves whether to participate, which included the right to opt out from the programme. Of those invited, 1885 adolescents (88 %) agreed to participate in the cohort with parental consent (figure 2).

**Table 1:** Sociodemographic characteristics of participants, % (n=1885)

Gender	
Female	49.2
Male	50.8
Foreign background	
Child or both parents	18.4
Perceived no. of siblings*, M (SD)	2.0 (1.3)
Child-rated household status, Wave 1	
Living with both birth parents	80.6
Living with one birth parent	7.8
Dual-residence	10.7
Other arrangements†	0.2
Children with any parent on welfare benefits, Wave 1‡§	
Any parent on welfare benefits	13.6
Children living in a household receiving welfare benefits, Wave 1‡	
Living with one birth parent on welfare benefits	1.3
Living with both birth parents on welfare benefits	1.1
Key outcomes	
Children referred to the Child Welfare Services from 2013 until age 18	20.4

Attrition varies due to different data sources and response rates.

* Including full siblings, half-siblings and stepsiblings.

† For example, foster parents.

‡ For example, unemployment insurance, social assistance benefits, sick-leave benefits.

§ Regardless of whether the child lives with that parent.

### Participant and public involvement statement

There was no participant or public involvement in the design or conduct of this study. Adolescents were involved in the construction of questionnaires via pilot testing which informed adaptation of the questionnaires so that they could accommodate adolescents with learning disabilities and attention deficits. During the LoRDIA and LoRDYA project period, a webpage was maintained to inform participants and the public on ongoing developments within the respective projects, including dissemination of results. A webpage is now being constructed for the Gothenland Millennium Cohort which will link to the LoRDIA and LoRDYA sites.

### Primary data collection

Comprehensive annual, self-report, pen and paper questionnaires containing 350–450 items per wave were used (a full list of variables can be made available on request). For the first three waves of data collection, both school grades in the cohort were surveyed simultaneously (Wave 1, 5 September 2013–15 November 2013, 12 and 13 year olds; Wave 2, 15 September 2014–15 December 2014, 13 and 14 year olds; Wave 3, 28 September 2015–6 November 2015, 14 and 15 year olds). In the fourth year of data collection (Wave 4, 6 October 2016–9 May 2017), only half of the cohort—the remaining 15-year-olds—were surveyed. Wave 5 (September 2017–9 May 2018 and 13 September 2018–9 February 2019) surveyed the two different age groups (during year 2 of upper secondary school when they were 17). Data were collected by pen and paper questionnaires in classrooms, supervised by the research team. Based on screening in Wave 5, 758 adolescents were invited to participate in structured clinical interviews in Wave 6. Interviews were conducted by interviewers via telephone (n=387; 51%) approximately 1 year following Wave 5 data collection. The interviewers were skilled in psychology or psychiatric nursing and trained in the chosen instruments. In addition to the adolescent assessments, parental questionnaires were administered in Waves 1 and 2, and teacher and school reports were administered in Waves 1–4.

#### Adolescent self-reports

In addition to administrative data, demographic background data and socioeconomic data, the questionnaires in Waves 1–5 covered psychological well-being and ill-health; puberty and body image; functional impairments and participation; personality; substance use and misuse; harassment and bullying (victimisation and perpetration); criminal behaviour; leisure time activities; parenting and parent-child relations; family and social factors; sibling, peer and romantic relations; experiences of childhood abuse and neglect; school relations and school functioning; contacts with helping professions and police. Peer network data, based on up to eight closest friends, were also collected. A personality assessment—the Junior version of the Temperament and Character Inventory (J-TCI)—was included in Wave 2; religion, sexual experiences, sexual orientation and sexual risk behaviours in Waves 3–5; educational plans, gender discontent and heredity in Wave 5. The structured clinical interviews in Wave 6 assessed more than 20 psychiatric diagnoses according to the 5th edition of the Diagnostic and Statistical Manual of Mental Disorders (DSM-5^23^) or the International Statistical Classification of Diseases and Related Health Problems (10th revision; ICD-10^24^), including various mood and anxiety disorders, eating disorders, psychosis, attention deficit hyperactivity disorder (ADHD) and attention deficit disorder, antisocial personality syndrome, various substance use disorders, as well as gambling and gaming disorders.

#### Pilots and adaptations to the adolescent self-reports

With the ambition to include all adolescents in the cohort, including those with learning disabilities and attention deficits, the questionnaires were adapted to be easy to understand and to complete. Therefore, questionnaires in all waves were piloted before use. *Read-aloud pilots* to 4–5 adolescents with and without learning difficulties were used to identify any problems youth had in understanding the questions in the survey. Based on this, wording was changed to make the surveys easy to understand and complete. *Full class pilots* (to both groups) of all scales, including those based on modified items, were checked for homogeneity and internal consistency. These showed, to our satisfaction, the same high psychometric quality after modification. Due to the positive experiences of using these adaptations, we decided to use the adapted questionnaires for all participants (ie, not only to those with learning difficulties) from Wave 2. This has been beneficial to the whole programme as it has made it easier for all participants to understand the questions and participate in the data collection over the years.

#### Validity and reliability of the adolescent self-reports

Most of the questions included in the questionnaires come from established and validated instruments with previously published psychometric properties. The psychometric properties (ie, homogeneity in factor analyses, internal consistency using Cronbach’s alpha) of all scales and waves were investigated during pilot testing and in conjunction with primary data collection for all waves. Internal consistency of measures based on collected data was found primarily to be in the range of satisfactory (>0.70) to excellent (>0.90). In addition, measure congruence has been investigated with long-term test-retests (ie, 1 year between the retests) with questions that refer to the same time-period across different waves.^25^,^27^ These tests showed that the test-retest stability was substantial or almost perfect for measures such as the Childhood Trauma Questionnaire (CTQ), witness to domestic violence, child welfare contact due to misconduct, police contact due to crime, age of onset for use of various substances and alcohol drunkenness. Substantial stability in long-term test-retest on sensitive items adds support to the validity of the self-reports. In addition, some areas of investigation use triangulation methods with multiple informants (eg, adolescents, parents) providing reports on the same issues (eg, J-TCI, and communication patterns in the family).^28 29^

#### Parental reports

Questionnaires were mailed to parents in Waves 1 and 2. In Wave 1, the questions concerned information about the adolescent and about the parents themselves. In total, 550 families replied. Parents were given the option of filling in separate forms or one form together. For those who filled in separate forms (n=256; that is, 128 pairs), there was acceptable agreement on questions about the adolescent between parents who lived together (n=102) but poor agreement when living separately (n=26). When combining two separate reports from parents, information about the adolescent was taken from the parents who lived together with the adolescent, including alternating residence, and coded as the mean of the two. Since Wave 2 only concerned information about the adolescent, one parental form was sent to the address where the adolescent lived and could be answered by one or both parents (n=759).

The Wave 1 parental questionnaire covered birth country of adolescent and parents; language(s) spoken at home; year of adoption when applicable; family structure, including living together, living with other, siblings, etc; education and employment of both parents; family accommodation; family economy; welfare support; child-parent connectedness; child-parent communication; parental control; parent management of peer relations; child defiance. The questionnaire also included questions intended to be answered separately by each parent including: parental self-efficacy; parental substance use and related problems; parental anxiety and depression. Wave 2 questionnaires included the Swedish parental version of the J-TCI^30^.

#### Teacher and school reports

Short web surveys were distributed annually during the spring term in Waves 1–4 to the teacher responsible for the class that year. The survey was estimated to take less than 10 min per participant to fill out. It surveyed the teachers’ knowledge about participants, their language skills in Swedish, various observed disabilities, school adaptations and support to meet special needs, ratings of engagement in and relation to school, and school functioning. In addition, the school administration provided information on whether participants studied Swedish as a second language and on school merits (grades in all subjects and attendance). In Wave 1, information was also provided on the participant’s curriculum (ordinary compulsory school or special needs compulsory school) and whether the participant received education in their mother tongue.

#### Attrition

Across the 1885 adolescents included in the cohort, 1760 (93.4%) participated in at least one data collection wave up to grade 9 (Wave 4) and there was 70–85% participation (depending on wave) per data collection wave. In Wave 5, about half (50.4%; n=949) of the adolescents participated. Attrition in Wave 5 was not equal across gender. Response rates were lower among boys (53.8%) than girls (41.3%, p<0.001). Comparisons based on national taxation registers found no differences between adolescents that participated in Wave 5 and those that did not participate in Wave 5 in foreign origin or in the extent to which households were living in relative poverty.^31^ Similarly, using questionnaire data collected in Wave 3, we found no differences in emotional health, psychosomatic problems, alcohol or drug use problems^31^ for those that participated in Wave 5 versus those that did not. There was, however, a difference in delinquent behaviours across groups. Participants in Wave 5 scored lower than non-participants on delinquent behaviours (p=0.007^31^). Across Waves 1–5, 96% of the study population participated in at least one data collection wave. The response rate for parental questionnaires was low (n=32%; 41%). In examining demographic differences among adolescents whose parents participated in the first wave parental questionnaire, we found no significant differences in adolescent gender (p=0.686) or subjective economic status (p=0.246). However, response rates were lower among parents of adolescents with non-foreign backgrounds (21.7% vs 32.4%, p<0.001) and among parents from separated families (28.9% vs 36.6%, p=0.006). Similarly, in the second wave, no differences emerged with respect to adolescent gender (p=0.380). Yet, participation was lower among parents of adolescents with non-foreign backgrounds (34.1% vs 42.5%, p<0.001), from separated families (32.0% vs 48.5%), and from families with both lower and higher subjective economic status compared with those reporting an average status (lower 36.5% vs average 46.8% vs higher 43.0%, p=0.013).

Attrition on teacher reports was <30% across Waves 1–4.

### Municipal child welfare services’ records

Data from municipal child welfare services’ (CWS) records for all adolescent participants were collected retrospectively at age 18 for all referrals and contacts with CWS for the period covering entry into the study to age 18. Data were collected from CWS in the four participating municipalities as well as from an additional 38 municipalities in which participants were found to have lived between the ages of 13 and 18. Data were collected regarding referrals, assessments, decisions on support interventions, provision of interventions, documented goal attainment and continued needs. Data collection was undertaken between 13 March 2019 and 26 November 2019. The researcher had access to identifying information during data collection.

### Population-based register data

Sweden offers many reliable data sources for register-based research, thanks to its population-based registers containing personal data. The system of unique personal identity numbers enables researchers to link data across various registers for specific individuals. Statistics Sweden is the government agency charged with collating and housing national statistics and Statistics Sweden linked register data from the following sources to the Gothenland Millennium Cohort data collected during Waves 1–5. At the time of linkage, Statistics Sweden removed all personal identity numbers from the data file and replaced them with non-identifiable codes. Statistics Sweden houses the original code key for the Gothenland Millenium Cohort which makes future linkages possible. Gothenland Millenium Cohort researchers do not have access to identifying information on individual study participants as relates to population-based register data or the merged data files.

#### National Board of Health and Welfare

Data from seven national registries are included: Cause of Death Register, Inpatient Register, Specialist Outpatient Register, Dental Health Register, Prescribed Drug Register, Register of Care for Substance Misuse and Register of Measures for Children and Young Persons. Data include information on causes of death, diagnoses, treatments, medicines prescribed and social welfare interventions. Data were requested on 29 November 2022.

#### Statistics Sweden

Data from two sources are included in the cohort data: the Longitudinal Integrated Database for Health Insurance and Labor Market Studies and the Education Register. Data include information on socioeconomic variables, school achievement, employment and income and labour market programme participation. Data were requested on 8 November 2022.

#### The Swedish National Council for Crime Prevention

Data were collected from two national registries, the Criminal Suspect Register and the Swedish Crime Register, which include information on socio-demographics, charges and arrests, sentencing and convictions. Data were requested on 29 November 2022.

#### The Swedish Defense Conscription and Assessment Agency

Data were also obtained from the Military Conscription Registry and the Swedish National Archives (for individuals drafted before 1996) which include dates of service, recruitment decisions and eligibility, as well as several health-related variables. Data were requested on 23 November 2022 and 26 January 2023 respectively.

## Funding

### Primary funding

Primary funding was granted in 2012 for the period 2013–2020 in a combined decision (No 259-2012-25) from four research foundations: Swedish Research Council (VR); Swedish Research Council for Health, Working Life and Welfare (FORTE); Sweden’s Innovation Agency (VINNOVA); and The Swedish Research Council Formas to initiate cohort data collection within the program, *Teenage development – A prospective longitudinal research program on young people’s social networks, addiction, mental health and school adaptation,* which was later changed to *LoRDIA*. New funding was obtained from FORTE (No 2019-00280) to extend data collection to national data registers and institutional records. Data within this wave of collection allows young people to be followed to age 20 within the project name LoRDYA.

### Secondary funding

Major and minor grants have been awarded in parallel to primary funding of research both within and outside of LoRDIA and LoRDYA. These include projects studying adolescents with disabilities (Sävstaholm Foundation no. ST-2014-023; Sunnerdahl Disability Foundation no. 40-14; Futurum Jönköping County no. 2014/3821-271), the Importance of the Parent-Child Relationship for Adolescents’ Attitudes, Gambling with or without Money - Knowledge and Prevention (FORTE ref. no. 2021–01696), What Happens to the Parent-Child Relationship when Adolescents Drink? (Swedish Retail Alcohol Monopoly’s Research Fund ref. no. FO2021-0070) and Gaming and Mental Health Among Young People in Sweden (FORTE ref. no. 2022–00143). Other studies include Children of Parents who Drink Excessively (Swedish Retail Alcohol Monopoly’s Research Fund, ref. no. 2019-0029), Bullying in Relation to Emotional Health among Students (Public Health Agency of Sweden, ref. no. 00751-2019-2.3.1), Risky Drinking – Developmental Patterns of Alcohol Use in Adolescence (Swedish Retail Alcohol Monopoly’s Research, ref. no. 2020–0065), and Healthy Schools an Arena for Promoting Good and Equal Health by Advancing Vulnerable Students’ Employment and Income Opportunities (FORTE ref. no. 2024–01608).

## Findings to date

### Publications

As of September 2025, the Gothenland Millenium Cohort and related projects have resulted in over 240 publications, including nine doctoral dissertations, two licentiate dissertations, more than 55 peer-reviewed publications, 35 student theses and 100+conference presentations. Additional publications include non-peer-reviewed papers, media presentations and reports. A full list of publications as of September 2025 is available as supplementary material.

### Disabilities and everyday life functioning

The International Classification of Functioning, Disability and Health defines participation as involvement in life situations and is closely linked to well-being. Research using Millennium Cohort data has highlighted the importance of using code-level rather than chapter-level coding for adolescent mental health and identified different types of participation, suggesting behaviors like risk-taking should be considered separately from other types of participation.^32^ Studies have also explored disability and participation, such as Lygnegård *et al*^33 34^ who examined factors affecting participation for adolescents with and without neurodevelopmental disorders (NDD). Family support and environment were key for higher participation, with participation profiles remaining stable over time.^35^ Gender was a stronger influence on participation than NDD. Carlberg *et al*^36^ noted disadvantaged situations for adolescents with self-reported NDD, while Augustine *et al*^37^ found that adolescents with NDD face more mental health challenges, but participation is more strongly related to well-being than NDD or mental health problems.

### Child-parent relationships

Research on parent-child relationships undertaken using Millennium Cohort data^38^,^40^ highlights that adolescent disclosure—when teens voluntarily share information with parents—is the strongest predictor of parental knowledge about adolescent activities. Strong parent-child bonds and high parental self-efficacy promote adolescent disclosure.^39^ While parental behavioral control (setting rules) is also linked to parental knowledge, active monitoring (parental solicitation) has less impact. Adolescent disclosure is protective against mental health issues and risk behaviours, while parental behavioural control helps prevent criminal behaviour.^41 42^ However, parental solicitation has no similar protective effects and can worsen outcomes, especially for teens with adventurous temperaments or those with poor parent-child relationships.^40 41^ Parents often overestimate both their knowledge and their children’s communication, with discrepancies linked to mental health problems: internalizing issues in girls and externalizing problems in boys.^29^

### Child welfare

Child welfare contact was explored in the cohort. By utilizing self-ratings in Waves 1 – 4, findings showed that every fourth child gave responses that indicated severe exposure to maltreatment (ie, child abuse, neglect) or severe behavioral problems (ie, substance misuse, criminal behaviour or other socially destructive behaviour) or both.^25^ Based on administrative data, about every fifth child is referred to Child Welfare Services.^43^ However, fewer than half of the children with self-reported indicators of severe exposure to maltreatment or behavioural problems were referred and, across all referrals, more than half of all children did not report any severe exposure. Factors related to socioeconomic vulnerabilities are important for understanding these referral processes, such as living with a single parent or living in families with low incomes. Taken together, these findings indicate that children with and without severe exposure to maltreatment or behavioural problems are equally likely to be referred to Child Welfare Services.

When referrals are assessed by Child Welfare Services, those concerning girls are more likely to be investigated than those concerning boys.^44^ The reasons for referrals have no explanatory power in understanding the decision to investigate girls, while referrals related to neglect and behavioural problems decrease the likelihood of investigation for boys. Girls are more likely to be investigated if they live in poverty or show, according to self-ratings, indicators of severe exposure to maltreatment and/or behavioral problems. Boys, on the other hand, are less likely to be investigated as they get older. Whether the child had previously been referred to Child Welfare Services predicted the likelihood of child welfare investigations for boys and girls.

### Substance use and criminal behaviour

Substance use was investigated in the cohort across several studies.^2628 31 45^,^49^ Longitudinal within-person analyses across adolescence found no relations between alcohol inebriation and illegal drug use, except early (age 13 or under) episodes of inebriation were linked to initiating drug use a year later.^48^ No associations were found between drug use and later criminality, although moderate relationships were found between criminality and later drug use. Three sub-groups of substance-using patterns were found across ages 13–16: abstainers, occasional users and regular users, highlighting heterogeneity in use.^49^ While an occasional substance use pattern was linked to higher novelty-seeking personality and more peers who use substances, the regular substance use pattern was linked to negative family cohesion at the start of adolescence, lower perceived relative Socioeconomic status (SES), as well as peer and personality factors.^49^ Tobacco use was associated with increased alcohol consumption, with high frequency and dual use of cigarettes and snus being more strongly linked to alcohol use.^45^

Gender differences in personality and links to alcohol use were also explored. Girls with low well-being and mental health problems were overrepresented among those with early alcohol experiences.^28^ Internalising problems were linked to alcohol inebriation for boys, which in turn was linked to low self-directedness.^46^ For girls, inebriation was linked to low harm avoidance. For both genders, the combination of low self-directedness and cooperativeness with high novelty seeking and low harm avoidance was a predictor of inebriation, both directly and indirectly, through mental health problems.^46^

Characteristics of non-drinking adolescents, with no previous experience with alcohol, differed from drinking adolescents in terms of fewer social interactions, less positive attitudes towards alcohol, a lower probability of using other drugs and fewer conduct problems, which may imply a more introverted personality function.^50^

Parent-child relationship quality was linked to adolescent alcohol use and inebriation. Adolescent disclosure, as opposed to parental solicitation, was linked to lower levels of inebriation at age 17.^51^ Increases in parental knowledge of their child’s whereabouts tended to be followed by lower levels of inebriation during early and mid-adolescence, though increases in inebriation in late adolescence were in turn linked to reductions in parental knowledge the following year.^47^ Parents’ own alcohol drinking was also found to be of significance for adolescents’ alcohol consumption, especially for 17-year-olds.^51^

The prevalence of Substance Use Disorders (SUD) at the age of 18 was estimated to be 14.6%. This mostly concerned dependence and harmful use of alcohol and was more frequent for girls than boys.^31^

### Mental health

Mental health and mental well-being have been investigated in the cohort.^28 29 31 40 46 52^ Externalizing problems, in contrast to internalizing problems, occurred more commonly in adolescents who reported a high degree of well-being.^28^ Good self-esteem in early adolescence tended to continue during adolescence and was also linked to having good perceived mental well-being. Normal levels of self-esteem, rather than high levels, at age 12 and 13 years were, however, linked to good wellbeing in later adolescence. Girls reported low self-esteem more often than boys.^52^ Parents’ overestimation of the level of parent-adolescent communication, including adolescent disclosure and parental solicitation in particular, was found to be disadvantageous for adolescent psychological health.^40^

Associations between mental health and personality traits were studied. Externalising problems were linked to high novelty-seeking and low self-directedness, whereas internalising problems were linked to high harm avoidance and low self-directedness.^46^ Based on structured psychiatric assessments, the prevalence of psychiatric disorders in the cohort was estimated at 26.7% for at least one of 16 psychiatric disorders, other than addictions, surveyed. These were twice as frequent in girls compared with boys, with depression being the most common disorder. Psychiatric comorbidity with SUD was found to be common in adolescents, and girls with SUDs were at higher risk of suffering from other psychiatric conditions.^31^

### Trauma

Childhood maltreatment and trauma were extensively studied within the cohort, using the CTQ, CTQ-Short Form.^53^ CTQ was validated within the cohort, showing substantial and almost perfect long-term test-retest reliability for all scales, acceptable convergent validity and moderate discriminatory ability.^27^ CTQ scales were also used when exploring self-rated severe exposure to maltreatment.^25^ Findings showed that emotional abuse and neglect are related to externalizing problems, internalizing problems, psychosomatic symptoms and mental well-being, in a dose-response pattern.^54^ Girls reported a larger increase in mental health problems at lower levels of emotional maltreatment than boys. School absenteeism was almost twice as common among children exposed to maltreatment.^55^ Maltreated children absent from school reported more mental health problems, experienced more bullying and had worse relationships with their teachers than other children absent from school. Maltreated children had a higher likelihood of experiencing negative consequences from using alcohol and/or illicit drugs compared with those without such experiences.^56^ This relationship was only weakly mediated by the quantity of alcohol and other illicit drugs consumed.

### Harassment and victimisation

Several studies have examined peer victimization, including sexual harassment, and its impact on adolescents. Risk factors for victimization include female gender, early pubertal timing^57^ and neurodevelopmental disabilities.^58^ Early puberty increases harassment risk due to mismatched cognitive and physical development.^59^ However, peer victimization and bullying tend to decrease as adolescents mature, especially for those with early pubertal timing. Skoog and Kapetanovic^60^ found a reciprocal relationship between sexual harassment and mental health issues. Sexual harassment led to increased mental health problems, which in turn exacerbated harassment the following year. This cycle was particularly strong in girls. Although sexual harassment declined when transitioning to high school, mental health impacts persisted. Skoog and Kapetanovic^61^ identified that supportive peer relationships helped mitigate the negative effects of sexual harassment on mental health. Strong bonds with classmates were protective, whereas teacher-student or parent-child relationships did not show the same effect. These findings emphasize the importance of promoting healthy peer dynamics and mental health support in school-based interventions.

### Sexual and gender minorities

Sexual orientation was examined through attraction patterns across three studies.^62^,^64^ Investigating the prevalence of sexual and gender minorities, the findings showed that at age 17 most girls (75.5%) and boys (88.3%) were identified as heterosexual. Bisexuality was notably more common among girls, with 18.2% identifying as bisexual compared with just 4.5% of boys. Homosexuality was reported for 2.6% of girls and 3.1% of boys. A smaller portion of the population was identified as non-sexual or asexual, with 3.7% of girls and 4.1% of boys falling into this category. Homosexual and bisexual orientations were, however, relatively unstable over time. Non-sexuality decreased with age, often shifting toward heterosexuality.^62^ Gender discontent was endorsed by 1.7% and was about the same across genders.^62^ Sexual orientation was found to be associated with distinct personality traits, which was particularly notable in girls.^63^ Specifically, girls identified as bisexual showed traits linked to emotional instability, while boys identified as bisexual had higher harm avoidance. Girls identified as asexual displayed a stable and content profile, while girls identified as homosexual scored lower on reward dependence.^63^ Moreover, girls identified as homosexual and adolescents identified as bisexual, regardless of gender, exhibited the highest rates of mental health disorders.^64^ Girls identified as bisexual showed significantly elevated risks for conditions such as depression, ADHD and substance use (alcohol and drugs), while boys identified as bisexual faced increased risks, particularly for suicidality, obsessive compulsive disorder (OCD), attention deficit hyperactivity disorder (ADHD) and antisocial personality disorder. Gender-discontent adolescents had moderately more psychiatric problems than gender-content adolescents. Interestingly, adolescents identified as asexual appeared to be healthier than other groups. Both the gender discontent and asexual groups had small sample sizes.^64^

## Future plans

The Gothenland Millennium Cohort is a valuable resource for examining well-being across the transition from adolescence into adulthood. It contains a rich array of multi-informant and intergenerational data which encompasses self-reports, parent reports, teacher reports, administrative and register data. Future plans include continued investigation of well-being and its related indicators from multiple perspectives during adolescence and transition from adolescence to adulthood. This includes plans to continue to secure funding and expand the data collection for longer-term follow-up.

### Strengths and limitations

The Gothenland Millenium Cohort data have many strengths. Due to their prospective longitudinal nature, their representativeness of a general adolescent population and the wide number of measures used, the data provide a powerful base for studying changes and development over time from early adolescence to adulthood across a number of areas important for individual wellbeing. In addition, cohort data includes multiple informants as well as population-based register data which allows for investigation from multiple perspectives. Due to the size of the cohort, subgroup analyses are possible. Despite these strengths, three limitations should be highlighted. First, as the cohort followed is from a general population, certain specific subgroups of adolescents may be small or not fully represented in the cohort. Second, not all measures used are included in every data collection wave, meaning that longitudinal analyses need careful planning in the cohort data. Third, attrition was high among certain groups (eg, parents in Wave 1 and Wave 2) and waves (eg, adolescent reports in Wave 5). In most studies reviewed here, attrition weighting was not conducted. Exceptions to this are the two studies on prevalence of psychiatric conditions, which were based on a two-phase design of screening in Wave 5 and diagnostic interview in Wave 6, and a strategy to handle interview attrition.^31 64^ (A supplement to the latter study presents exact calculations of the formulas that can be used in similar studies in the future.) Future studies using data from waves and respondents in which there is high attrition should consider weighting. Weighting can help reduce bias by making the remaining sample more representative and preserving sample size, which is positive for statistical power and subgroup analysis.^65^ However, weighting can disproportionately influence study results and typically assumes that attrition is Missing at Random.^66^ These, and other weighting issues, should be carefully considered prior to applying weights to the cohort data in upcoming studies.^67^

## Supplementary material

10.1136/bmjopen-2025-100327 online supplemental file 1

## Data Availability

No data are available.
