# Supplementary material for: The Swedish longitudinal Gothenland Millennium Cohort for studying wellbeing from early adolescence through adulthood
Source: BMJ Open. 2025 Dec 10;15(12):e100327. doi: 10.1136/bmjopen-2025-100327 (PMC12699670; doi:10.1136/bmjopen-2025-100327)
Supplement: online supplemental file 1 [file bmjopen-15-12-s001.docx]

# Publications from LoRDIA and LoRDYA based on The Gothenland Millenium Cohort

## September 2025

## PhD Dissertations

1. Torbjörn Kalin (2024). Om upptäckt och sortering till social barnavård. Longitudinella perspektiv på barn som far allvarligt illa. Jönköping University, School of Health and Welfare.
2. Kristina Carlén (2022). Predictors of mental health in adolescents – with a salutogenic perspective. Jönköping University, School of Health and Welfare.
3. Olov Aronsson (2021). Understanding [the social integration of adolescents of foreign origin. Longitudinal investigations of inter-origin friendship formation](https://www.researchgate.net/publication/353982404_Understanding_the_social_integration_of_adolescents_of_foreign_origin_Longitudinal_investigations_of_inter-origin_friendship_formation). Jönköping University, School of Health and Welfare.
4. Russell Turner (2020). Teenage kicks. The differential development of drug use, drunkenness, and criminal behaviour in early to mid-adolescence. University of Gothenburg, Department of Social Work.
5. Johan M. Hagborg (2020). Child maltreatment among young adolescents – effects on mental health, academic functioning, and substance use. University of Gothenburg, Department of Psychology.
6. Sabina Kapetanovic (2019). Mutual actions – Developmental links between aspects of the parent-adolescent relationship and adolescent risk behaviors. Jönköping University, School of Health and Welfare.
7. Frida Lygnegård (2018). Participation in and outside school: Self-ratings by Swedish adolescents with and without impairments and long-term health conditions. Jönköping University, School of Health and Welfare.
8. Karin Boson (2018). Psychological Perspectives on Alcohol Use Among Young Adolescents – Mental Health and Personality. University of Gothenburg, Department of Psychology.
9. Birgitta Ander (2018). Ungdomars berusningsdrickande – Vem, var och med vilka? Jönköping University, School of Health and Welfare.

## Licentiate dissertations

1. Johan M. Hagborg (2018). Child maltreatment and its association with school factors and mental health in early adolescence. University of Gothenburg, Department of Psychology.
2. Karin Boson (2016). How do the “tweenies” do? Mental health, alcohol experiences and personality among young adolescents. University of Gothenburg, Department of Psychology.

## Peer Reviewed articles

1. Arne Gerdner, Therése Skoog, Sabina Kapetanovic, Emma Claesdotter-Knutsson, Susanna Askelöf & Anders Håkansson (2025-10, in press). Distribution of mental health diagnoses in relation to sexual orientation and gender discontent in a late adolescent community population. *BMC Psychiatry*.
2. Emma Claesdotter-Knutsson, Sabina Kapetanovic, Therése Skoog, Susanna Askelöf, Anders Håkansson, and Arne Gerdner (2025). Prevalence of Sexual and Gender Minorities in a Swedish Adolescent Community Population; Stability and Fluidity of Sexual Orientations. *Archives of Sexual Behavior.* https://doi.org/10.1007/s10508-025-03226-y
3. Therése Skoog, Emma Claesdotter-Knutsson, Anders Håkansson, Sabina Kapetanovic, and Arne Gerdner (2025). Sexual Orientation and Personality Dimensions among Adolescents Measured by the Junior Temperament and Character Inventory. *Archives of Sexual Behavior*, 54:2321–2332. DOI: https://doi.org/10.1007/s10508-025-03180-9
4. Russell Turner, Ylva Bjereld & Lilly Augustine (2025). Developmental Relations Between Peer Victimization, Emotional Symptoms, and Disability/Chronic Condition in Adolescence: Are Within- or Between-Person Factors Driving Development? *Journal of Youth and Adolescence* 54:1063–1078. <https://doi.org/10.1007/s10964-024-02114-3>
5. Johanna Andersson, Kristina Berglund, Robin Irmel, & Louise Adermark (2024). Prospective Association Between Tobacco Use and at-Risk Alcohol Consumption Among Swedish Adolescents: Outlining the Influence of Tobacco Product, Frequency of Use and Gender in the LoRDIA Cohort. *Tobacco Use Insights* 17: 1–10. DOI: 10.1177/1179173X241298524
6. Torbjörn Kalin (2024). Examining the likelihood of being referred to the Child Welfare Services in Sweden. Influences at the individual, peer, and environmental levels. *Child & Family Social Work*, 0:1–11. <https://doi.org/10.1111/cfs.13248>
7. Karin Boson, Sabina Vlasman, & Kristina Berglund (2024). When a non-drinker continues to be a non-drinker during adolescence. A reply to Raninen et al. *International Journal of Adolescence and Youth*, *29*(1). https://doi.org/10.1080/02673843.2024.2379688
8. Karin Boson, Sevtap Gurdal, Emma Claesdotter‑Knutsson, & Sabina Kapetanovic (2024). Adolescent gaming and parent–child emotional closeness: bivariate relationships in a longitudinal perspective. *Current Psychology.* https://doi.org/10.1007/s12144-024-05714-1
9. Karin Boson, Sabina Vlasman, & Kristina Berglund (2024). Characteristics of non-drinking adolescents: a longitudinal Swedish study. *International Journal of Adolescence and Youth*, 29(1). <https://doi.org/10.1080/02673843.2024.2312861>
10. Sabina Kapetanovic & Russell Turner (2024). Interplay between Parental Knowledge and Adolescent Inebriation, and Their Links to Parent-Child Relationships over Time [*Youth*](https://www.researchgate.net/journal/Youth-2673-995X) 4(1):163-176. DOI: [10.3390/youth4010012](http://dx.doi.org/10.3390/youth4010012)
11. Kristina Carlén, Sakari Suominen, & Lilly Augustine (2023). The association between adolescents’ self-esteem and perceived mental well-being in Sweden in four years of follow-up. *BMC Psychology,* 11(1), 1–413. [https://doi.org/10.1186/s40359-023-01450-6](https://eur02.safelinks.protection.outlook.com/?url=https%3A%2F%2Fdoi.org%2F10.1186%2Fs40359-023-01450-6&data=05%7C02%7CArne.Gerdner%40ju.se%7C00ee636d604748a9c93708dc53219e81%7C7564bc8f37384b4dbd575a02ca6215fb%7C0%7C0%7C638476651060065392%7CUnknown%7CTWFpbGZsb3d8eyJWIjoiMC4wLjAwMDAiLCJQIjoiV2luMzIiLCJBTiI6Ik1haWwiLCJXVCI6Mn0%3D%7C0%7C%7C%7C&sdata=72RtTGrrjJFcFlYX94bkKnhs%2BQFbOMSt7FjF4a%2BxJ84%3D&reserved=0)
12. Kristina J. Berglund, Karin Boson, Peter Wennberg, & Arne Gerdner (2022). Impacts of alcohol consumption by mothers and fathers, parental monitoring, adolescent disclosure, and novelty-seeking behaviour on the likelihood of alcohol use and inebriation among adolescents. *International Journal of Adolescence and Youth.* <https://doi.org/10.1080/02673843.2022.2156298>
13. Torbjörn Kalin, Thorbjörn Ahlgren, & Birgitta Persdotter (2022). Gender disparities in child welfare services' assessments of referrals. Findings from Sweden. *Child Abuse & Neglect*, 134: 105918. <https://doi.org/10.1016/j.chiabu.2022.105918>.
14. Arne Gerdner & Anders Håkansson (2022). Prevalence and comorbidity in a Swedish adolescent community sample – gambling, gaming, substance use, and other psychiatric disorders. *BMC Psychiatry,* 22: 594. DOI: 10.1186/s12888-022-04218-1
15. Johan M. Hagborg, Torbjörn Kalin, & Arne Gerdner (2022). The Childhood Trauma Questionnaire - Short Form (CTQ-SF) used with adolescents – methodological report from clinical and community samples. *Journal of Child and Adolescent Trauma.* DOI: 10.1007/s40653-022-00443-8
16. Torbjörn Kalin, Thorbjörn Ahlgren, Birgitta Persdotter, & Arne Gerdner (2022). How do Child Welfare Referrals match the self-reports of Severely Exposed Children in Sweden? [*Child & Family Social Work*](https://onlinelibrary.wiley.com/journal/13652206), [*27*(2](https://onlinelibrary.wiley.com/toc/13652206/2022/27/2)): 100–111. (Wileys utnämnde denna till “top cited article” både 2022 och 2023 ). <https://doi.org/10.1111/cfs.12856>
17. Russell Turner (2022). Getting real about youth substance use and crime: how ‘realistic’ theories can improve knowledge and understanding for practice. *British Journal of Social Work*, 52, 396–415. DOI: 10.1093/bjsw/bcaa244. Advance Access Publication January 26, 202152.
18. Therese Skoog & Sabina Kapetanovic (2022). The intertwined evolution of sexual harassment victimization and emotional problems among young people. *Journal of Social Issues.* [https://doi.org/10.1111/josi.12514](https://eur02.safelinks.protection.outlook.com/?url=https%3A%2F%2Fdoi.org%2F10.1111%2Fjosi.12514&data=05%7C02%7CArne.Gerdner%40ju.se%7C732da5c42d3d4be45fec08dc53ac2658%7C7564bc8f37384b4dbd575a02ca6215fb%7C0%7C0%7C638477246045695090%7CUnknown%7CTWFpbGZsb3d8eyJWIjoiMC4wLjAwMDAiLCJQIjoiV2luMzIiLCJBTiI6Ik1haWwiLCJXVCI6Mn0%3D%7C0%7C%7C%7C&sdata=Wk5WWK3%2BBceQhRe8YEzAaQrx%2FbV6Am4FdwQ50NSUEwc%3D&reserved=0)
19. Lilly Augustine, Frida Lygnegård, & Mats Granlund (2022). Trajectories of participation, mental health, and mental health problems in adolescents with self-reported neurodevelopmental disorders. *Disability and rehabilitation, 44*(9), 1595-1608. DOI: 10.1080/09638288.2021.1955304.
20. Frida Lygnegård, Mats Granlund, Sabina Kapetanovic, & Lilly Augustine (2021). Short-term longitudinal participation trajectories related to domestic life and peer relations for adolescents with and without self-reported neurodevelopmental impairments. *Heliyon 7(4):*  e06784. [DOI: 10.1016/j.heliyon.2021.e06784](https://doi.org/10.1016/j.heliyon.2021.e06784)
21. Thorbjörn Ahlgren, Torbjörn Kalin & Arne Gerdner (2021). Self-rated child maltreatment, behavioural problems, and contacts with welfare and police authorities – longitudinal community data. *European Journal of Social Work, 24*(4): 642-656*.* DOI: [10.1080/13691457.2021.1896996](https://doi.org/10.1080/13691457.2021.1896996)
22. Therese Skoog & Sabina Kapetanovic (2021). The Role of Pubertal Timing in the Development of Adolescent Peer Victimization and Offending. *Developmental Psychology, 42*(1):5-32*.* [DOI: 10.1177/02724316211002265](https://doi.org/10.1177%2F02724316211002265)
23. Lilly Augustine, Frida Lygnegård, Margareta Adolfsson & Mats Granlund (2021). The utility of the International Classification of Functioning construct as a statistical tool – operationalizing mental health as an indicator of adolescent participation. *Disability and Rehabilitation, 44*(16): 4220-4226. DOI: [10.1080/09638288.2021.1884295](https://doi.org/10.1080/09638288.2021.1884295)
24. Sabina Kapetanovic & Therese Skoog (2021). The Role of the Family’s Emotional Climate in the Links between Parent-Adolescent Communication and Adolescent Psychosocial Functioning. *Research on Child and Adolescent Psychopathology, 49*:141–154. DOI: [10.1007/s10802-020-00705-9](https://www.researchgate.net/deref/http%3A%2F%2Fdx.doi.org%2F10.1007%2Fs10802-020-00705-9?_sg%5B0%5D=ZaLN5WvDAgtPhkK-1BPC1GcsyzHgp6I3hOzwaWEEIEf3ZlMKZ6gLGOM6WgeIhLqOAU3yG8sN8m3aYCEdLbjIgA4dUw.JTJ3pnyNCBLD82h5R3bnfPRINECvHI2vCnuAXldlTkzQCrCB2vglOlNlJAK4XyNWj513N4saWgzRcPJsq6orGQ)
25. Olov Aronson & Arne Gerdner (2020). Youth centers, structured leisure activities, and friends of native and foreign origin: A short-term longitudinal study. *Journal of Leisure Research*, *52*(3):265–285. DOI: [10.1080/00222216.2020.1780521](https://doi.org/10.1080/00222216.2020.1780521)
26. Sabina Kapetanovic & Karin Boson (2020). Discrepancies in Parents’ and Adolescents’ Reports on Parent-Adolescent Communication and Associations to Adolescents’ Psychological Health. *Current Psychology,* 1-12. DOI: [10.1007/s12144-020-00911-0](https://www.researchgate.net/deref/http%3A%2F%2Fdx.doi.org%2F10.1007%2Fs12144-020-00911-0?_sg%5B0%5D=aZRbpRAlhb_B6LO0QLie6XoCts2UAbDlmdZUcObdBo80H6ksUdxR7DAiOhfPRqW_igW0F-3gvG41tcsI3FpgZRe6Mg.XrKc-gQoeqE_S5pxizxum40Haa_AlfEU0Q4oV20vQtp-Z6RF6pFAYkImHb2_CbZa3DnCaR4hUJkjj-OCakq94g)
27. Therese Skoog & Sabina Kapetanovic (2020). The Role of Relational Support in the Longitudinal Links between Adolescent Sexual Harassment Victimization and Psychological Health. *Development and Psychopathology*, 1-13. DOI: [10.1017/S0954579420000565](https://doi.org/10.1017/S0954579420000565)
28. Johan M. Hagborg, Valgeir Thorvaldsson & Claudia Fahlke (2020). Child maltreatment and substance-use-related negative consequences: Longitudinal trajectories from early to mid-adolescence. [*Addictive Behaviors*](https://www.sciencedirect.com/science/journal/03064603), *106*: 106365. [DOI: 10.1016/j.addbeh.2020.106365](https://doi.org/10.1016/j.addbeh.2020.106365)
29. Birgitta Ander, Eleonor Fransson, Disa Bergnehr & Arne Gerdner (2020). Onset in the use of tobacco, alcohol, and drugs in early adolescence. *Journal of Social Work Practice in the Addictions,* *20*(2): 105–121. [DOI: 10.1080/1533256X.2020.1748973](https://doi.org/10.1080/1533256X.2020.1748973)
30. Sabina Kapetanovic, Therése Skoog, Margareta Bohlin & Arne Gerdner (2020). Does one Size Fit All?—Linking Parenting with Adolescent Substance Use and Adolescent Temperament. *Journal of Research on Adolescence,* [*30* (S2](https://onlinelibrary.wiley.com/toc/15327795/2020/30/S2)): 443-457.  [DOI: 10.1111/jora.12489](https://doi.org/10.1111/jora.12489)
31. [Russell Turner](https://www.sciencedirect.com/science/article/pii/S0306460319303983?casa_token=w7F2qqzxAz8AAAAA:jqfSxFdt-1iL9fR2Uvn_f6Yd8E9AAHdUqeAi22eAdGdfynMPGf08J_9Ffw9GXFSwYDhJKSk0b-g#!), [Kristian Daneback](https://www.sciencedirect.com/science/article/pii/S0306460319303983?casa_token=w7F2qqzxAz8AAAAA:jqfSxFdt-1iL9fR2Uvn_f6Yd8E9AAHdUqeAi22eAdGdfynMPGf08J_9Ffw9GXFSwYDhJKSk0b-g#!) & [Anette Skårner](https://www.sciencedirect.com/science/article/pii/S0306460319303983?casa_token=w7F2qqzxAz8AAAAA:jqfSxFdt-1iL9fR2Uvn_f6Yd8E9AAHdUqeAi22eAdGdfynMPGf08J_9Ffw9GXFSwYDhJKSk0b-g#!) (2020). Explaining trajectories of adolescent drunkenness, drug use, and criminality: A latent transition analysis with socio-ecological covariates. [*Addictive Behaviors*](https://www.sciencedirect.com/science/journal/03064603)*,* [*102*](https://www.sciencedirect.com/science/journal/03064603/102/supp/C), 106145. [DOI: 10.1016/j.addbeh.2019.106145](https://doi.org/10.1016/j.addbeh.2019.106145)
32. Sabina Kapetanovic, Margareta Bohlin, Therese Skoog & Arne Gerdner (2020; on-line 2017): Structural relations between sources of parental knowledge, feelings of being overly controlled and risk behaviors in early adolescence. *Journal of Family Studies,* *26*(2): 226-242. DOI: [10.1080/13229400.2017.1367713](https://doi.org/10.1080/13229400.2017.1367713)
33. Sabina Kapetanovic, Savannah Boele & Therése Skoog (2019). Parent-Adolescent Communication and Adolescent Delinquency: Unraveling Within-Family Processes from Between-Family Differences. *Journal of Youth and Adolescence,* *48*: 1707–1723. [DOI: 10.1007/s10964-019-01043-w](https://link.springer.com/article/10.1007%2Fs10964-019-01043-w)
34. Berit Møller Christensen, Maria Björk, Lena Almqvist & Karina Huus. (2019). Patterns of support to adolescents related to disability, family situation, harassment, and economy. *Child: Care, Health and Development,* [*45*(5](https://onlinelibrary.wiley.com/toc/13652214/2019/45/5)): 644-653. [DOI: 10.1111/cch.12675](https://doi.org/10.1111/cch.12675)
35. Karin Boson, Peter Wennberg, Claudia Fahlke & Kristina Berglund (2019), Personality traits as predictors of early alcohol inebriation among young adolescents: Mediating effects by mental health and gender-specific patterns. *Addictive Behaviors,* [*95*](https://www.sciencedirect.com/science/journal/03064603/95/supp/C): 152-159. [DOI: 10.1016/j.addbeh.2019.03.011](https://doi.org/10.1016/j.addbeh.2019.03.011)
36. [Louise Carlberg](https://journals.sagepub.com/doi/full/10.1177/1403494818788415?casa_token=4uj86KrtXmsAAAAA%3AubwZzOaG_QkNym-q081rDgfIq8oZ_lsidZLbk8kVgpFh20NTP8NJvWHHj24srqm1EZmPvSx2PxfpTA) & [Mats Granlund](https://journals.sagepub.com/doi/full/10.1177/1403494818788415?casa_token=4uj86KrtXmsAAAAA%3AubwZzOaG_QkNym-q081rDgfIq8oZ_lsidZLbk8kVgpFh20NTP8NJvWHHj24srqm1EZmPvSx2PxfpTA) (2019). Achievement and participation in schools for young adolescents with self-reported neuropsychiatric disabilities: A cross-sectional study from the southern part of Sweden. *Scandinavian Journal of Public Health*, *47* (2): 199-206. [DOI: 10.1177/1403494818788415](https://doi.org/10.1177%2F1403494818788415)
37. Sabina Kapetanovic, Therese Skoog, Margareta Bohlin & Arne Gerdner (2019). Aspects of the Parent–Adolescent Relationship and Associations with Adolescent Risk Behaviors Over Time. *Journal of Family Psychology, 33*(1): 1-11. Advance online publication, 2018. [DOI: 10.1037/fam0000436](http://dx.doi.org/10.1037/fam0000436)
38. [Frida Lygnegård](http://www.frontiersin.org/people/u/508895), [Lilly Augustine](http://www.frontiersin.org/people/u/515814), [Mats Granlund](http://www.frontiersin.org/people/u/345841), [Ingemar Kåreholt](http://www.frontiersin.org/people/u/553022) & Karina Huus (2018). Factors Associated with Participation and Change Over Time in Domestic Life, Peer Relations, and School for Adolescents With and Without Self-Reported Neurodevelopmental Disorders. A Follow-Up Prospective Study. *Frontiers in Education, 3*: 28*.* Frontiers Media SA. <https://doi.org/10.3389/feduc.2018.00028>
39. Frida Lygnegård, Lena Almqvist, Mats Granlund & Karina Huus (2018): Participation profiles in domestic life and peer relations as experienced by adolescents with and without impairments and long-term health conditions. *Developmental Neurorehabilitation, 22*(1): 27-38. <https://doi.org/10.1080/17518423.2018.1424266>
40. Russell Turner, Kristian Daneback & Anette Skårner (2018). Assessing reciprocal association between drunkenness, drug use, and delinquency during adolescence: Separating within- and between-person effects. *Drug and Alcohol Dependence*, [*191*](https://www.sciencedirect.com/science/journal/03768716/191/supp/C): 286-293. [DOI: 10.1016/j.drugalcdep.2018.06.035](https://doi.org/10.1016/j.drugalcdep.2018.06.035)
41. Lilly Augustine, Frida Lygnegård, Mats Granlund & Margareta Adolfsson (2018): Linking youths’ mental, psychosocial, and emotional functioning to ICF-CY: lessons learned. *Disability and Rehabilitation, 40*(19): 2293-2299. [DOI: 10.1080/09638288.2017.1334238](https://doi.org/10.1080/09638288.2017.1334238)
42. Johan M. Hagborg, Kristina Berglund & Claudia Fahlke (2017). Evidence for a relationship between child maltreatment and absenteeism. *Child Abuse & Neglect,* *75*; 41-49. [DOI: 10.1016/j.chiabu.2017.08.027](https://doi.org/10.1016/j.chiabu.2017.08.027)
43. Johan M. Hagborg, Inga Tidefors & Claudia Fahlke (2017). Gender differences in the association between emotional maltreatment with mental, emotional, and behavioral problems in Swedish adolescents. [*Child Abuse & Neglect*](https://www.sciencedirect.com/science/journal/01452134)*,* [*67*](file:///\\ad.hj.se\files\Staff\Restricted\HHJ_LoRDIA_Admin\Delstudier\Översikt%20över%20LoRDIA-publikationer\67):249-259. <https://doi.org/10.1016/j.chiabu.2017.02.033>
44. Karin Boson, Sven Brändström & Sören Sigvardsson (2017). The Junior Temperament and Character Inventory (JTCI): Psychometric Properties of Multi-Informant Ratings. *Psychological Assessment, 30*(4), 550–555. [DOI: 10.1037/pas0000513](https://psycnet.apa.org/doi/10.1037/pas0000513)
45. Karin Boson, Kristina Berglund, Peter Wennberg & Claudia Fahlke (2016). Well-Being, Mental Health Problems, and Alcohol Experiences Among Young Swedish Adolescents: A General Population Study. *Journal for Person-Oriented Research,* *2*(3): 123-134. [DOI: 10.17505/jpor.2016.12](https://doi.org/10.17505/jpor.2016.12)

## Preprints

1. Karin Boson, Sabina Kapetanovic, Peter Wennberg, Kristina Berglund (2025). Adolescent temperament and parental alcohol provision predict adolescent hazardous alcohol use. Preprint in Research Square https://doi.org/10.21203/rs.3.rs-7206982/v1
2. Arne Gerdner, Therése Skoog, Sabina Kapetanovic, Emma Claesdotter-Knutsson, Susanna Askelöf & Anders Håkansson (2024). Distribution of mental health diagnoses in relation to sexual orientation and gender dysphoria in a late adolescent community population. Preprint in Research Square <https://doi.org/10.21203/rs.3.rs-3958877/v1> (See the published version above under slightly different name).

## Articles pending

1. Johanna Andersson, Kristina Berglund, Robin Irmel, Louise Adermark (2025, submitted). Trajectories in Youth Mental Health in relation to Tobacco Use – Outlining gender differences in prospective and cross-sectional associations in the LoRDIA programme.
2. Johan Melander Hagborg & Valgeir Thorvaldsson (manuscript). Impact of child maltreatment on the developmental trajectories of three domains of academic functioning in adolescence.
3. Tina M. Olsson, Torbjörn Kalin, Sabina Kapetanovic, Russell Turner, Arne Gerdner (2025). Cohort Profile: The Swedish longitudinal Gothenland Millennium Cohort for studying wellbeing from early adolescence through adulthood. BMJ Open (revised)
4. Johan Melander Hagborg & Torbjörn Ahlgren (manuscript). Associations between child maltreatment and criminal justice involvement in emerging adulthood.
5. Johan Melander Hagborg & Russell Turner (manuscript). The role of temperament as a mediator between child maltreatment and alcohol/drug use in adolescence*.*
6. Arne Gerdner, Therése Skoog, Emma Claesdotter-Knutsson, Anders Håkansson, Sabina Kapetanovic (to be revised). Contextual, Predictive, and Subsequential Factors of Sexual Debut before the Legal Age of Consent – a Prospective Longitudinal Community Study.
7. Diana Kajic & Torbjörn Kalin (2023, submitted). Social bonds to school are related to school merits and changes over time. A longitudinal study.
8. Kristina Berglund, Karin Boson, Peter Wennberg, Sabina Kapetanovic (2024, submitted). Can adolescents' temperament exacerbate the risk of future hazardous alcohol consumption when parents offer them alcohol? (Manuscript)
9. Sabina Kapetanovic & Arne Gerdner (to be revised). Raised under Parental Dominance of Adolescent’s Romantic Relations within a Modern Welfare State.
10. Torbjörn Kalin & Thorbjörn Ahlgren (manuscript). Low levels of participation in structured leisure-time activities among emotionally neglected children.
11. Russell Turner (manuscript). The role of early debut, parents, and peers in adolescent alcohol use and ‘risky’ drinking.

## Chapters in anthologies

1. Birgitta Persdotter & Torbjörn Kalin (2023). Att upptäcka och ge stöd till barn som riskerar att fara illa. In: Torbjörn Forkby, Sofia Enell & Johanna Thulin (red). *Att komma rätt i rätt tid – kritiska frågor för kunskapsbaserad prevention till barn och unga*. Lund: Studentlitteratur. p. 197-214.
2. Torbjörn Forkby, Russell Turner & Kristina Alstam (2020). Den strukturerade fritiden – en möjlighet till lärande för livet? In: Alireza Behtoui, Fredrik Hertzberg, Anders Neergaard (Red.), *Ungdomars Fritidsaktiviteter*. Lund: Studentlitteratur, p. 37-58.

## Articles in dissertations

1. Russell Turner (2020). Personality, parents or peers? The differential development of teenage drunkenness, drug use, and criminal behaviour: a multi-level exploration using socio-ecological covariate. Published in: Russell Turner: *Teenage kicks. The differential development of drug use, drunkenness, and criminal behaviour in early to mid-adolescence.* University of Gothenburg, Department of Social Work
2. Olov Aronson (2021). Digital leisure and interethnic friendships: A longitudinal study Published in: Olov Aronsson, *Understanding* [*the social integration of adolescents of foreign origin. Longitudinal investigations of inter-origin friendship formation*](https://www.researchgate.net/publication/353982404_Understanding_the_social_integration_of_adolescents_of_foreign_origin_Longitudinal_investigations_of_inter-origin_friendship_formation). Jönköping University, School of Health and Welfare.
3. Olov Aronson, Disa Bergnehr & Michael Wells (2021). Parents, friendship formation, and subjective wellbeing: A longitudinal study of adolescents with native and migrant backgrounds. Published in: Olov Aronsson, *Understanding* [*the social integration of adolescents of foreign origin. Longitudinal investigations of inter-origin friendship formation*](https://www.researchgate.net/publication/353982404_Understanding_the_social_integration_of_adolescents_of_foreign_origin_Longitudinal_investigations_of_inter-origin_friendship_formation)*.* Jönköping University, School of Health and Welfare.
4. Birgitta Ander, Disa Bergnéhr, Eleonor I. Fransson & Arne Gerdner (2018). Where and with whom – contexts of 15-year-olds’ drunkenness (Manuscript published in: Ander, B. *Ungdomars berusningsdrickande – Vem, var och med vilka?* Jönköping University. School of Health and Welfare).

## Master theses

1. Sabina Vlasman (2022). The Non-drinking Adolescents – What Characterizes them based on Psychological Health, Social relations, and Attitudes towards Alcohol? Göteborgs Universitet, Psykologiska Institutionen.
2. Karl Mogren (2021). Subjective well-being among adolescents and young adults with substance use problems. Specialist thesis in clinical psychology, Department of Psychology, University of Gothenburg.
3. Anna Gustavsson (2021). Faktorer som påverkar skoltrivsel hos gymnasieelever med neuropsykiatrisk funktionsnedsättning. Institutionen för beteendevetenskap och lärande. Linköpings Universitet.
4. Diana Kajic (2021). Sociala band och skolframgångar. En kvantitativ studie om sambandet mellan sociala band till skolan och avgångsbetyg i årskurs 9. Jönköping University, School of Health and Welfare, Department of Social Work.
5. Lotte Sophie Moes (2021). The relationship between mental health in adolescents having self-reported neurodevelopmental disorders and sources of parental knowledge: A cross-sectional study. Jönköping University, School of Education and Communication.
6. Nina Ambrus (2021). Utsatthet för barnmisshandel och relationer till jämnåriga i ungdomsåren. Göteborgs Universitet, Psykologiska Institutionen.
7. Sara Mehdin & Lise Mueller (2019). Barnmisshandel och dess påverkan på sexuell hälsa och sexuellt risktagande bland unga i Sverige. Göteborgs Universitet, Psykologiska Institutionen
8. Torbjörn Kalin (2018). Fritidsvanor hos socialt utsatta barn. Jönköping University. School of Health and Welfare, Department of Social Work.
9. Qi Meng (2018). School’s social environment in relation to participation and well-being of young adolescents with self-rated neurodevelopmental problems. Jönköping University, School of Education and Communication.
10. Katarina Olsson (2018). Ungdomars upplevelser av påverkan i relation till vuxna: En fenomenologiskt inspirerad studie utifrån ett agens och empowerment-perspektiv University West, Department of Social and Behavioural Studies, Division of Psychology, Pedagogy and Sociology.
11. Maria Löfgren (2017). Pojkars och flickors våldsbeteende, alkohol- och drogkonsumtion och psykiska hälsa. Göteborgs Universitet, Psykologiska Institutionen.
12. Daniel Ryding (2017). Sociodemographic risk factors for emotional maltreatment in Swedish adolescents. Göteborgs Universitet, Psykologiska Institutionen.
13. Louise Carlberg (2016). Participation in schools for young adolescents with neuropsychiatric disabilities: A cross-sectional study from the Southern part of Sweden. Uppsala Universitet, Department of Women’s and Children’s Health
14. Stefan Roosch Haraldsson & Carl Cederqvist (2015). Adolescents’ Experience of Parental Reactions and its Relations to Externalizing and Internalizing Problems. Göteborgs Universitet, Psykologiska Institutionen
15. Johanne Svantorp (2015). En studie av elevers deltakelse i skolen. Masteroppgave i spesialpedagogikk. Institutt for spesialpedagogikk, Det utdanningsvitenskapelige fakultet, Universitetet i Oslo

## Candidate theses

1. Therése Isteri och Elin Josefsson (2023). Mobbning i relation till symtom på psykisk ohälsa – En longitudinell studie om förhållandet mellan ungdomars självskattade utsatthet av mobbning och upplevelse av symtom på psykisk ohälsa. Jönköping University, School of Health and Welfare, Department of Social Work.
2. Timea Jakobsson & Alexandra Möllerström (2022). Syskons band i våldets spår: En tvärsnittsstudie av våldsutsatta barns självskattade kvalitet i syskonrelationer. Jönköping University, School of Health and Welfare, Department of Social Work.
3. Jennie Andersson & Anna Skoglund (2021). Utsatthet under barndomen och upplevelse av förälder som trygg hamn. Göteborgs Universitet, Psykologiska institutionen.
4. Almqvist, Rebecka & Mascarenhas, Emilia (2021). Sambandet mellan ungdomars föräldra- och kompisrelationer. En studie om hur ungdomars upplevda föräldrastöd påverkar nivån av stöd och konflikt i deras närmaste kompisrelation. Jönköping University, School of Health and Welfare, Department of Social Work.
5. Tobias Abrahamsson & Hossein Matin (2020). Familjestruktur, föräldrainsyn och vänners inverkan på ungdomsbrottslighet – En kvantitativ jämförelsestudie mellan resultat från LoRDIA och BRÅ:s skolundersökning rörande risk- och skyddsfaktorer för brott. Jönköping University, School of Health and Welfare, Department of Social Work.
6. Samuel Johansson & Lennart Lilja (2020). Barn till förälder med substansproblematik – Personlighetsegenskaper och substansproblematik. Jönköping University, School of Health and Welfare, Department of Social Work.
7. Christian Andréasson & Linnéa Engholm (2020). Den religiösa gemenskapens roll för skolungdomars välbefinnande. Jönköping University, School of Health and Welfare, Department of Social Work.
8. Sara Berggren & Maria Stenered (2020). Kroppsuppfattning hos ungdomar i årskurs 8 och 9. Jönköping University, School of Health and Welfare, Department of Social Work.
9. Jenny Karlsson & Kerstin Arvidsson (2019). Vardagskommunikation och sexuella riskbeteenden. Jönköping University, School of Health and Welfare, Department of Social Work.
10. Josefine Tarberg & Annica Wedenhjelm (2019). Psykosomatiska symtom. En studie om barns upplevda tillit till föräldrar i förhållande till psykosomatiska symtom. Jönköping University, School of Health and Welfare, Department of Social Work.
11. Rebecca Strand & Frida Rösberg (2019). Internaliserade problem hos ungdomar i årskurs nio.: En studie med tvärsnittsdesign som tittar på internaliserade problem relaterat till mobbning och föräldraskap. Jönköping University, School of Health and Welfare, Department of Social Work.
12. Sylvia Karlsson & Emma Björk (2018). Psykisk ohälsa hos barn på glesbygden: En kvantitativ enkätstudie. Jönköping University, School of Health and Welfare, Department of Social Work.
13. Cia Skog & Leif Ternström (2018). Erhåller traumatiserade ungdomar professionell kontakt – och hur mår de? Psykologiska Institutionen, Göteborgs Universitet.
14. David Bothén & Jarkko Tauriainen (2017). Ungdomsbrottslighet - Risk och skydd i familjerelationer. Jönköping University, School of Health and Welfare, Department of Social Work.
15. Malin Englaborn & Mika Mörner (2017). Det är lättare att bli kriminell än att skaffa ett jobb - en studie om ungdomsbrottslighet och möjlighetshorisonter. Trollhättan: Högskolan Väst.
16. Alexander Alterot & Mattias Toivainen (2017). Alkoholbruk hos ungdomar i årskurs 8 och 9. Påverkan genom influenser från föräldrar och vänner. Jönköping University; School of Health and Welfare, Department of Social Work, Department of Social Work.
17. Timmy Johansson, Josefine Klasson & Marlene Mardo (2017). Typiskt pojkar: om psykisk hälsa, maskulinitet och normbrytande beteende bland unga pojkar. Jönköping University, School of Health and Welfare, Department of Behavioural Science and Social Work.
18. Susanne Engelbrektsson & Joakim Turtell (2017). Vilka ungdomar mobbar andra? Samband med impulsivitet, våldsbenägenhet och föräldrabarnkommunikation. University West, Department of Social and Behavioural Studies, Division of Psychology, Pedagogy and Sociology.
19. Anna Karlsson & Charlotte Lindén (2017). Vad får ungdomar att begå brott? - Undersökning om samband mellan ungdomsbrottslighet och personlighetsdrag, Familjerelationer, Vänners brottsliga beteende, bostadsområde och kön. Trollhättan: Högskolan Väst.
20. Sara Andersson & Caroline Sandström (2016). Att mobba andra. En studie om mobbning, psykisk hälsa, tobak, alkohol och droganvändning och föräldrabarnkommunikation. Trollhättan: Högskolan Väst.

## Research presentations

### International level

1. Russell Turner (2022). Teenage kicks? When to worry about teenage alcohol/drug use and crime. Paper presentation at the 18th Biennial Conference of the European Association for Research on Adolescence (EARA), August 24-27, 2022, Dublin, Irland.
2. Karin Boson (2022). Impacts of parental alcohol consumption and monitoring, adolescent disclosure and novelty-seeking on adolescents’ alcohol use – should we worry? Paper presentation at the 18th Biennial Conference of the European Association for Research on Adolescence (EARA), August 24-27, 2022, Dublin, Irland.
3. Torbjörn Kalin (2022). Criminal behaviour, substance misuse and other socially destructive behaviour – did someone worry? Paper presentation at the 18th Biennial Conference of the European Association for Research on Adolescence (EARA), August 24-27, 2022, Dublin, Irland.
4. Torbjörn Kalin (2022). Children referred to the CWS. Factors that lead to decisions to investigate. Presentation at European Conference for Social Work Research (ECSWR), Amsterdam.
5. Sabina Kapetanovic & Therese Skoog (2020). The Role of Family Emotional Climate in the Links between Parent-Adolescent Communication and Adolescent Psychological Problems. Paper presentation at the 17th Biennial Conference of the European Association for Research on Adolescence (EARA), September 2-5, 2020, Porto, Portugal.
6. Therese Skoog & Sabina Kapetanovic (2020). The Role of Pubertal Timing in the Development of Adolescent Peer Victimization and Offending. Paper presentation at the 17th Biennial Conference of the European Association for Research on Adolescence (EARA), September 2-5, 2020, Porto, Portugal.
7. Therese Skoog & Sabina Kapetanovic (2020). The Role of Social Support in the Longitudinal Link between Adolescent Sexual Harassment and Psychological Health [Paper presentation]. Society for Research on Adolescence Conference, San Diego, California, USA. (March 19-22, Conference cancelled due to pandemic.)
8. Sabina Kapetanovic & Karin Boson (2019). Differences in parents' and adolescents' reports on parental knowledge and longitudinal associations to adolescents' psychological problems. Presentation at ECDP - European conference on developmental psychology - Aten, Grekland 29/8- 1/9, 2019
9. Johan Hagborg (2019). Child Maltreatment and Alcohol Related Negative Consequences – longitudinal trajectories from early to mid-adolescence. International Society for Traumatic Stress Studies 35th annual meeting, Boston
10. Therese Skoog (2018). Multiple aspects of challenge to adolescent growth: Presentations from the Swedish LoRDIA project. Chair and organizer of a research symposium at the 16th Biennial Conference of the European Association for Research on Adolescence, Ghent, Belgium.
11. Russell Turner (2018). Re-thinking ‘risk’ in the context of nurturing adolescent growth – What can we learn from reciprocal associations between traditional teenage ‘risk’ behaviours? Oral presentation within symposium: Multiple aspects of challenge to adolescent growth: Presentations from the Swedish LoRDIA project. European Association of Research on Adolescence (EARA), Ghent.
12. Karin Boson (2018). Predicting alcohol use and inebriation through mental health problems, well-being and personality dimensions among young adolescents. Oral presentation within symposium: Multiple aspects of challenge to adolescent growth: Presentations from the Swedish LoRDIA project. European Association of Research on Adolescence (EARA), Ghent.
13. Sabina Kapetanovic (2018). Bidirectional Links between Adolescent Disclosure, Parental Solicitation, Parental Control and Adolescent Delinquency. Oral presentation within symposium: Multiple aspects of challenge to adolescent growth: Presentations from the Swedish LoRDIA project. European Association of Research on Adolescence (EARA), Ghent.
14. Johan M. Hagborg (2018). “Child Maltreatment and School Absenteeism”. Oral presentation within symposium: Multiple aspects of challenge to adolescent growth: Presentations from the Swedish LoRDIA project. European Association of Research on Adolescence (EARA), Ghent
15. Russell Turner (2018). Improving longitudinal quantitative research with adolescents – 7 lessons from LoRDIA. Presentation to Substance Use and Addiction (SUAB) European network meeting, Tilburg.
16. Sabina Kapetanovic (2017). Parenting and adolescent risk behaviours. Presentation at European Conference on Developmental Psychology (ECDP), Utrecht
17. Johan M. Hagborg (2017). Gender Differences in the Association Between Emotional Maltreatment with Mental, Emotional, and Behavioral Problems in Swedish Adolescents”. The 15th European Society for Traumatic Stress Studies Conference, Odense.
18. Karin Boson (2017). Emotional, Psychological and Social Well-being: The Applicability of Mental Health Continuum Short Form (MHC-SF) among Young Adolescents. Poster presentation at the 5th World Congress in Positive Psychology, International Positive Psychology Association (IPPA) 13-17 Juli 2017, Montreal, Canada.
19. Karin Boson (2016). The Swedish Version of Junior Temperament and Character Inventory (JTCI): Psychometric Properties of Children’s Self-Report and Caregivers’ Rating. Presentation at XV Biennial conference, European Association for Research on Adolescence (EARA), La Barrosa, Cádiz, Spain.
20. Russell Turner (2016). Kids, drugs, and delinquency: Differential patterns and developmental pathways in adolescence. Presentation at European Association for Research on Adolescence (EARA), La Barossa, Spain.
21. Karin Boson (2016). The Swedish Version of Junior Temperament and Character Inventory (JTCI): Psychometric Properties of Children’s Self-Report and Caregivers’ Rating” vid European Conference on Personality Psychology (ECP) Paper presentation at The European Association for Personality Psychology (EAPP), Timisoara, Romania.
22. Sabina Kapetanovic (2016). Capturing parental knowledge and adolescent risk behaviour. Presentation at European Association for Research on Adolescence (EARA), La Barossa, Cádiz, Spain.
23. Karin Boson (2016). Well-being, mental health problems, and alcohol experiences among young Swedish adolescents: a general population study. Presentation at KBS 2016 - 42nd Annual Alcohol Epidemiology Symposium of the Kettil Bruun Society, Stockholm
24. Karin Boson (2016). Well-being, mental health problems, and alcohol experiences among young Swedish adolescents: a general population study. ISBRA ESBRA World Congress on Alcohol and Alcoholism, Berlin. Poster presentation.

### Nordic or national level

1. Sabina Kapetanovic (2025). “Does relational support play a role for the effect of sexual harassment on adolescent mental health?” Presentation at the national research seminar “Young People and Sexuality: Research and Practice”. January 24, 2025; Gothenburg University.
2. Johan M. Hagborg (2025). “Impact of child maltreatment on the developmental trajectories of three domains of academic functioning in adolescence.” Presentation at the national research seminar “Young People and Sexuality: Research and Practice”. January 24, 2025; Gothenburg University.
3. Torbjörn Kalin (2021). Referrals to the Child Welfare Services. Are the severely exposed children referred? Presentation på Nordic Network on Longitudinal Child Welfare Research (NORDLOCH). Spring seminar.
4. Torbjörn Kalin (2021). Upptäcker vi samma barn? Om samhällets förmåga att identifiera barn som far illa, om kognitiva genvägar och osäkra bedömningssituationer. Presentation på Förbundet för Forskning i Socialt arbete (FORSA) Café 2021 (online).
5. Torbjörn Kalin (2021). LoRDYA. Longitudinal Research on Development to Young Adults – A prospective longitudinal research programme aimed to study adolescent development from early adolescence into adulthood (emerging adulthood). Presentation på Nasjonalt Utviklingssenter for Barn og Unge (NUBU), Oslo 2021
6. Torbjörn Kalin (2020). Referral rates to the child welfare services. Relations between severe exposure of child maltreatment and behavioural problems and referrals to the child welfare services. Presentation på den tredje nationella barnavårdskonferensen, 1-2 dec. (On-line).
7. Thorbjörn Ahlgren (2020). Barn som är svårt utsatta enligt självskattning – övergrepp, försummelse och beteendeproblem samt förutsättningar för upptäckt. [Children who are severely exposed according to self-assessment – abuse, neglect, and behavioural problems as well as conditions for detection.] Presentation på Tredje Barnavårdskonferensen, 1-2 dec. (On-line).
8. Sabina Kapetanovic (2020). Föräldraskap och ungas utveckling. Presentation i samband med utdelande av ”Juniora priset”, Systembolaget, 27 nov. (On-line).
9. Olov Aronsson (2018). Predicting young immigrants’ native friends, immigrant friends, and social interactions: A short-term longitudinal study. Paper presented at Nordic Migration Research Conference, REMESO, Norrköping, 15-17 August, 2018.
10. Russell Turner (2018). Re-thinking ‘risk’ – ungdomar, alkohol och droger. Vad kan vi lära oss från reciprok risk för alkohol- och drogbruk och kriminalitet under tonåren? Presentation till Sonad, Stockholm.
11. Birgitta Ander (2016). Early onset in use of alcohol, tobacco and drugs. Presentation at Nordic Alcohol and Drug Researchers' Assembly (NADRA) in Helsinki.
12. Birgitta Ander (2016). Early onset in use of alcohol, tobacco and drugs. Presentation at Nordic Youth Research Symposium (NYRIS13), Trollhättan
13. Birgitta Ander (2016). The importance of place for adolescent binge drinking Presentation at Nordic Youth Research Symposium (NYRIS13), Trollhättan
14. Birgitta Ander (2016). "I can then dance like John Travolta." Binge drinking and the importance of places. Presentation at NordAN (Nordic Alcohol and Drug Policy Network), Oslo
15. Frida Lygnegård (2016). Involving Students with Intellectual disabilities in a longitudinal Study-experiences from the Swedish Research Programme LoRDIA. Presentation at Nordic Youth Research Symposium (NYRIS), Trollhättan.
16. Sabina Kapetanovic (2016). Structural relationships between parental knowledge and adolescent risk behaviors. Presentation at Nordic Youth Research Symposium (NYRIS), Trollhättan.
17. Russell Turner (2016). Kids, drugs, and delinquency: Differential patterns and developmental pathways in adolescence. Presentation at Paper Conference in Social Work, Lund.
18. Johan M. Hagborg (2016). The impact of psychological maltreatment on mental health in Swedish 12-13-year olds. Nordisk Förening mot barnmisshandel och Omsorgssvikt, Stockholm.
19. Karin Boson (2015). Well-being, mental health problems, and alcohol experiences among young Swedish adolescents: a general population study. Presentation at Swedish Association of Alcohol and Drug Research (SAD).
20. Karin Boson; Anette Skårner & Arne Gerdner (2014). Longitudinal Research on Development in Adolescence – LoRDIA. Presentation på Vetenskapsrådet (VR), Stockholm

### Regional or local level

1. Russell Turner (2015). Re-thinking ‘risk’ kring ungdomar, alkohol, droger och kriminalitet . Psychosocial research mental health in relation to alcohol and drug use in the development from adolescents to adults Presentation at CERA (Centre for Research and Education on Addiction), Gothenburg
2. Johan M. Hagborg (2015). The impact of psychological maltreatment on mental health in Swedish 12-13 year olds. Psychosocial research mental health in relation to alcohol and drug use in the development from adolescents to adults Presentation at CERA (Centre for Research and Education on Addiction), Gothenburg.
3. Karin Boson (2015). Välbefinnande, psykisk ohälsa och alkoholerfarenheter hos 12-13 åringar. Psychosocial research mental health in relation to alcohol and drug use in the development from adolescents to adults Presentation at CERA (Centre for Research and Education on Addiction), Gothenburg.

## Articles – not peer-reviewed

1. Johan M Hagborg (2024) har intervjuats i IOGT-NTO rapport om barn som växer upp i missbruk <https://www.junis.se/rapport/>
2. Lillebø, P-A., Almelid Vikenes, H. (2024, April 17). Foreldrene viktige for ungdommene. IOGT Region Midt-Norge*.*[https://idag.no/debattinnlegg/foreldre-viktige-for-ungdomane/19.45468](https://eur02.safelinks.protection.outlook.com/?url=https%3A%2F%2Fidag.no%2Fdebattinnlegg%2Fforeldre-viktige-for-ungdomane%2F19.45468&data=05%7C02%7CArne.Gerdner%40ju.se%7Ce8a9e7001d7046fa4d3f08dc62a7c29c%7C7564bc8f37384b4dbd575a02ca6215fb%7C0%7C0%7C638493719877432915%7CUnknown%7CTWFpbGZsb3d8eyJWIjoiMC4wLjAwMDAiLCJQIjoiV2luMzIiLCJBTiI6Ik1haWwiLCJXVCI6Mn0%3D%7C0%7C%7C%7C&sdata=1gA7uEz%2F6so5opOAsMGlMFok7BmOb3SLy1Ku%2BiIMhSU%3D&reserved=0)
3. Ringlund, O. M. (2024, March 12). Ungdom som ikke drikker har et nærere forhold til foreldrene sine. Høgskolen Innlandet. <https://www.inn.no/forskning/forskningsnyheter/ungdom-som-ikke-drikker-alkohol/>
4. Karin Boson (2024) har intervjuats på hemsidan för forskning.no: [Ungdom som ikke drikker, har et annet forhold til foreldrene sine (forskning.no)](https://eur02.safelinks.protection.outlook.com/?url=https%3A%2F%2Fwww.forskning.no%2Falkohol-og-narkotika-barn-og-ungdom-hogskolen-i-innlandet%2Fungdom-som-ikke-drikker-har-et-annet-forhold-til-foreldrene-sine%2F2334190&data=05%7C02%7CArne.Gerdner%40ju.se%7C335f46958824424dc1eb08dc6063c949%7C7564bc8f37384b4dbd575a02ca6215fb%7C0%7C1%7C638491229262895245%7CUnknown%7CTWFpbGZsb3d8eyJWIjoiMC4wLjAwMDAiLCJQIjoiV2luMzIiLCJBTiI6Ik1haWwiLCJXVCI6Mn0%3D%7C0%7C%7C%7C&sdata=BF48%2F%2F6WjhRB6n0T2%2BVuTj1%2FGeoxTA%2BiWpo0ZDBvu6Q%3D&reserved=0)
5. Maria Jernberg (2023). Intervju med Kristina Berglund: ”Föräldrarnas måttliga drickande påverkar barnet”. Psykologtidningen nr 2, 2023, s.32.
6. Frida Lygnegård (2019). ”Ungdomars röster om egen delaktighet.” Forskarintervju i Specialpedagogiska skolmyndighetens tidning Lika värde, nr 1, 2019. Länk: <https://www.spsm.se/stod/forskning-och-utveckling/aktuellt-om-forskning/nyheter/ungdomars-roster-om-egen-delaktighet/>
7. Frida Lygnegård (2019). Artikel om avhandlingen i Sveriges arbetsterapeuters tidning ”Arbetsterapeuten nr 3, 2019. Länk: [http://online.fliphtml5.com/tkwd/bliv/index.html#p=1](https://eur02.safelinks.protection.outlook.com/?url=http%3A%2F%2Fonline.fliphtml5.com%2Ftkwd%2Fbliv%2Findex.html%23p%3D1&data=02%7C01%7CArne.Gerdner%40ju.se%7Cd5e50b6a9b8648bcfd8208d7f0f73c8a%7C7564bc8f37384b4dbd575a02ca6215fb%7C0%7C0%7C637242816977403454&sdata=%2Bk6n8mz4duPcVvt6Ew7QZUIyvAQXfMzA6DmLO3yJ8rg%3D&reserved=0)
8. Frida Lygnegård (2019). Artikel om avhandlingen i den finska Arbetsterapeuttidningen
9. Frida Lygnegård (2019). Artikel om avhandlingen i tidskriften ”Omtanke” som ska spegla verksamhet inom Socialtjänst, vård och omsorg.
10. Frida Lygnegård (2019). Ungdomars röster om egen delaktighet Reportage i Tidningen Specialpedagogik (utges av Lärarförbundet) nr 1, 2019.
11. Sabina Kapetanovic (2018). ”Mindre riskbeteende med nära föräldrabarnrelation”. Skolhälsan: Stöd till Pojkar. Riksföreningen för Skolsköterskor
12. Frida Lygnegård (2018). ”Många faktorer styr elevers känsla av delaktighet”, Reportage till Skolporten (bevakar relevant forskning i syfte att stärka dialogen mellan forskare och lärare), länk: [https://www.skolporten.se/forskning/intervju/manga-faktorer-styr-elevers-kansla-av-delaktighet/](https://eur02.safelinks.protection.outlook.com/?url=https%3A%2F%2Fwww.skolporten.se%2Fforskning%2Fintervju%2Fmanga-faktorer-styr-elevers-kansla-av-delaktighet%2F&data=02%7C01%7CArne.Gerdner%40ju.se%7Cd5e50b6a9b8648bcfd8208d7f0f73c8a%7C7564bc8f37384b4dbd575a02ca6215fb%7C0%7C0%7C637242816977393457&sdata=%2BRPIYwOrMRHvb019T8gENv7Li5%2B8zwk4kwph6AdXuaY%3D&reserved=0)
13. Frida Lygnegård (2018). “Participation in and outside school: Self-ratings by Swedish adolescents with and without impairments and long-term health conditions” Forskningsnyhet på Skolporten publicerat 2018-09-26 länk: [https://www.skolporten.se/forskning/avhandling/participation-in-and-outside-school-self-ratings-by-swedish-adolescents-with-and-without-impairments-and-long-term-health-conditions/](https://eur02.safelinks.protection.outlook.com/?url=https%3A%2F%2Fwww.skolporten.se%2Fforskning%2Favhandling%2Fparticipation-in-and-outside-school-self-ratings-by-swedish-adolescents-with-and-without-impairments-and-long-term-health-conditions%2F&data=02%7C01%7CArne.Gerdner%40ju.se%7Cd5e50b6a9b8648bcfd8208d7f0f73c8a%7C7564bc8f37384b4dbd575a02ca6215fb%7C0%7C0%7C637242816977403454&sdata=OTYD%2BrqXQ9jWNEAtd6nm%2BlxOCfVrp3IUGtsbDQ78p0A%3D&reserved=0)
14. Frida Lygnegård (2018). ”Delaktighet i vardagen, ungdomar med och utan olika funktionsnedsättningar berättar” Reportage i Jönköping University´s tidning Me&JU, nr 4 2018
15. Karin Boson (2017). Forskning om ungdomar og alkoholbruk. Intervju för den fristående nyhetssidan Beernews.se i december 2017. Länk: <https://www.beernews.se/articles/karin-prisad-forskning-om-unga/>
16. Karin Boson (2017). Intervju om varför unga börjar dricka. Intervju både på web og trykk for IOGT-Sveriges medlemsblad Accent Länk: <http://www.accentmagasin.se/forskning/hon-undersoker-varfor-unga-borjar-dricka/>
17. Karin Boson, Andrea Malešević, Sabina Gušić, & Anders Wahlberg (2015). Psykologer: Regeringen kränker barns rättigheter. Svenska Dagbladet: Debattartikkel publisert både på web og tryck. Publicerad 2015-11-27. Länk: <https://www.svd.se/psykologer-regeringen-kranker-barns-rattigheter>
18. Frida Lygnegård (2015).”Lika, olika – Forskarporträtt i Sveriges arbetsterapeuters tidning”, Arbetsterapeuten, nr 2, 2015. Länk: [https://www.arbetsterapeuterna.se/media/1713/nr2_2015_lag.pdf](https://eur02.safelinks.protection.outlook.com/?url=https%3A%2F%2Fwww.arbetsterapeuterna.se%2Fmedia%2F1713%2Fnr2_2015_lag.pdf&data=02%7C01%7CArne.Gerdner%40ju.se%7Cd5e50b6a9b8648bcfd8208d7f0f73c8a%7C7564bc8f37384b4dbd575a02ca6215fb%7C0%7C0%7C637242816977403454&sdata=l38Tsb5HZUpIaV2NSAD4QcVzvcHqXmCutB3DcMzDPEQ%3D&reserved=0)
19. CERA (2015). ”Från molekylnivå till samhällsnytta” – Dokumentation från konferens Aktuell forskning om alkohol och narkotika, 8 oktober 2015. Dokumentationen innehåller referat av Karin Bosons, Johan Melander Hagborgs och Russell Turners presentationer. [https://www.lansstyrelsen.se/download/18.2c30d6f167c5e8e7c012de8/1547559205746/2015-48.pdf](https://eur02.safelinks.protection.outlook.com/?url=https%3A%2F%2Fwww.lansstyrelsen.se%2Fdownload%2F18.2c30d6f167c5e8e7c012de8%2F1547559205746%2F2015-48.pdf&data=04%7C01%7CArne.Gerdner%40ju.se%7C2aa166135ae8471da80308d8d69be5f1%7C7564bc8f37384b4dbd575a02ca6215fb%7C0%7C1%7C637495312376553219%7CUnknown%7CTWFpbGZsb3d8eyJWIjoiMC4wLjAwMDAiLCJQIjoiV2luMzIiLCJBTiI6Ik1haWwiLCJXVCI6Mn0%3D%7C1000&sdata=hINmFnrgDHlsj7XuGkqf6rbBu%2BQDtEICWPq0SkKVp6Y%3D&reserved=0)

## Presentations at conferences for practitioners and the public

### International level

1. Frida Lygnegård (2018). Patterns of participation within domestic life and peer relations - Youth’s own experiences. Oral presentation at World congress for Occupational Therapists in Cape Town, South Africa.

### Nordic or national level

1. Torbjörn Kalin (2022). Att upptäcka barn som far illa. Från barnens självskattade upplevelser till utredningar hos socialtjänsten. Presentation för Socialstyrelsen 2022 (online).
2. Birgitta Persdotter & Torbjörn Kalin (2022). Vilka barn upptäcks och varför? Om oro för barns hemförhållanden, barns självrapporterade utsatthet och professionellas agerande över tid. Presentation vid Socionomdagarna 2022, Stockholm.
3. Arne Gerdner (2019). Presentation av LoRDIA – Longitudinal Research on Development In Adolescence. Fokusspår: Att vara ung – jubileumssymposium om psykosociala faktorers påverkan på utvecklingen från barn till ung vuxen
   Presentation på Förebygg.NU, Göteborg 2019-11-14
4. Birgitta Ander (2019). Ungdomars berusningsdrickande – vem, var och med vilka? Fokusspår: Att vara ung – jubileumssymposium om psykosociala faktorers påverkan på utvecklingen från barn till ung vuxen. Presentation på Förebygg.NU, Göteborg 2019-11-14
5. Olov Aronson & Arne Gerdner (2019), Social kontext och vänskap för ungdomar med invandrarbakgrund. Fokusspår: Att vara ung – jubileumssymposium om psykosociala faktorers påverkan på utvecklingen från barn till ung vuxen
   Presentation på Förebygg.NU, Göteborg 2019-11-14
6. Karin Boson (2019). Hur hänger psykisk hälsa, personlighet och alkoholdrickande i tidiga tonår ihop? Fokusspår: Att vara ung – jubileumssymposium om psykosociala faktorers påverkan på utvecklingen från barn till ung vuxen. Presentation på Förebygg.NU, Göteborg 2019-11-14
7. Russell Turner (2019). Ungdomars riskbeteenden vad gäller alkohol, droger och kriminalitet. Var finns risken? Fokusspår: Att vara ung – jubileumssymposium om psykosociala faktorers påverkan på utvecklingen från barn till ung vuxen. Presentation på Förebygg.NU, Göteborg 2019-11-14
8. Sabina Kapetanovic (2019). Relationen mellan barn och föräldrar. Fokusspår: Att vara ung – jubileumssymposium om psykosociala faktorers påverkan på utvecklingen från barn till ung vuxen. Presentation på Förebygg.NU, Göteborg 2019-11-14
9. Birgitta Lager och Maria Holmstrand (2019). Exempel från Härryda. Fokusspår: Att vara ung – jubileumssymposium om psykosociala faktorers påverkan på utvecklingen från barn till ung vuxen. Presentation på Förebygg.NU, Göteborg 2019-11-14
10. Russell Turner (2017). Droger, alkohol och kriminalitet – Vad är kopplingen under tonåren? Presentation på Nationella Mässan - Förebygg.NU, Göteborg
11. Karin Boson (2017). Ungdomars psykiska hälsa och personlighet. Vad kan påverka tidigt alkoholbruk? Presentation på Nationella Mässan - Förebygg.NU, Göteborg.
12. Sabina Kapetanovic (2017). Föräldrar, tonåringar och tonåringars riskbeteende. Presentation på Nationella Mässan - Förebygg.NU, Göteborg
13. Birgitta Ander (2017). Var och med vilka? Ungdomars berusningsdrickande. Presentation på Nationella Mässan - Förebygg.NU, Göteborg

### Regional or municipal level

1. Torbjörn Kalin (2021). Upptäcker vi samma barn? Om samhällets förmåga att identifiera barn som far illa, om kognitiva genvägar och osäkra bedömningssituationer. Presentation för utvecklingsledare inom Kommunal utveckling, Jönköping.
2. Torbjörn Kalin (2021). Upptäcker vi samma barn? Om samhällets förmåga att identifiera barn som far illa, om kognitiva genvägar och osäkra bedömningssituationer. Presentation för chefsnätverket för chefer inom den sociala barnavården i Jönköpings län, Gränna.
3. Torbjörn Kalin & Birgitta Ander (2020). Att vara forskare och forska om barn. Presentation för elever vid Erik Dahlbergsgymnasiet i samband med ForskarFredag 2020 (online).
4. Torbjörn Kalin (2020). Att vara forskare och forska om barn. Presentation för elever vid Njudungsgymnasiet i samband med ForskarFredag 2020 (online).
5. Torbjörn Kalin & Thorbjörn Ahlgren (2020): Longitudinell forskning om barn och ungdom. Rön och planer för fortsatta studier. Presentation för Socialnämnden i Härryda, 2020-02-05.
6. Torbjörn Kalin & Thorbjörn Ahlgren (2019): Longitudinell forskning om barn och ungdom. Rön och planer för fortsatta studier. Presentation för Socialförvaltningens medarbetarkonferens i Härryda, 2019-10-16.
7. Russell Turner (2019). Att förebygga ungdomars riskbeteenden vad gäller alkohol, droger och kriminalitet: Vad ska vi göra? Presentation till Västra Götalands ANDTS samordnares träff. Göteborg 2019-11-22
8. Karin Boson (2017). Factors affecting adolescents' living conditions; well-being, mental health and alcohol use. Presentation at Expo Medica, Addiction Day, Stockholm
9. Johan M. Hagborg (2017). Maltreated children - who are they and what do they need to be successful in school? Two presentations at Härryda municipal teachers conference.
10. Johan M. Hagborg (2017). Children and adolescents with traumatic experiences - theory and practice. Presentation at University West, BUV (Barn- och ungdomsvetenskap) Conference Children and adolescents – research and practice in the same room, Trollhättan.
11. Arne Gerdner (2017). Could anyone become addicted? Addiction development, models of explanation and treatment strategies. Presentation at Regional Conference of Swedish Association of Alcohol and Drug Research (SAD), Umeå.
12. Birgitta Ander (2017), Where and with Whom? Adolescent binge drinking. Presentation at European Researchers’ Night, Jönköping.
13. Johan M. Hagborg (2017). From child maltreatment to substance use. Explanatory models. Two presentations at Open Conference, APEC (Addiction Psychology Experimental and Clinical Research) at Department of Psychology, Gothenburg University, Gothenburg.
14. Birgitta Ander & Mats Granlund (2016). Children and adolescents in Härryda municipality: Results from LoRDIA. Three presentations at Härryda municipal teachers conference.
15. Karin Boson (2016). Adolescents' personalities - risk and protection factors connected to mental health and addiction Presentation at Expo Medica, Addiction Day, Stockholm
16. Karin Boson (2016). Factors affecting adolescents' living conditions; wellbeing, mental health and alcohol use, Presentation at Research and Development North East (FoU Nordost), Stockholm
17. Sabina Kapetanovic (2016). Let me talk with you – adolescent risk behaviour and communication with their parents. Dalsland's Folk High School in Trollhättan, Three presentations at “Coffee break with researchers”. Three occations: Lysekil, Sotenäs and Trollhättan
18. Sabina Kapetanovic (2015). What do tweenies do and how do we reach them? Presentation at European Researchers' Night, Trollhättan.
19. Sabina Kapetanovic (2015). What do tweenies do and how do we reach them? Presentation at Researchers’ Grand Prix, Stockholm.
20. Karin Boson (2015). Hur trivs du i stort sett med livet? Psykiskt välbefinnande och ohälsa bland våra yngsta tonåringar. Presentation at ANDT (Alcohol, Drugs, Doping, Tobacco) Seminar, County Board, Jönköping
21. Karin Boson (2014). Föreläsning om preliminära resultat från forskningsprogrammet LoRDIA – Hur mår tweenisarna? vid konferens arrangerad av forskargruppen: Addiction Psychology: Experimental and Clinical research (APEC) at Department of Psychology, Gothenburg University, Gothenburg
22. Johan M. Hagborg (2014). Emotional neglect in adolescence. Consequences for mental health. Two presentations at Open Conference, APEC (Addiction Psychology Experimental and Clinical Research) at Department of Psychology, Gothenburg University, Gothenburg.
23. Karin Boson (2014). Presentation om ungdomars psykiska hälsa och alkoholbruk vid seminarium om ungdomar, föräldrar och alkohol, i arrangemang av Systembolaget i Malmö.
24. Karin Boson (2014). Föreläsning om personlighet vid personaldag for administrativ personal vid Psykologiska institutionen, Göteborgs universitet.

## Podcast & You-tube presentations

1. Kristina Berglund (2024-03-08). Unga som dricker mindre har färre kompisar. Radioinslag P4 Göteborg. [https://sverigesradio.se/artikel/forskaren-unga-som-inte-dricker-har-farre-vanner](https://eur02.safelinks.protection.outlook.com/?url=https%3A%2F%2Fsverigesradio.se%2Fartikel%2Fforskaren-unga-som-inte-dricker-har-farre-vanner&data=05%7C02%7CArne.Gerdner%40ju.se%7C335f46958824424dc1eb08dc6063c949%7C7564bc8f37384b4dbd575a02ca6215fb%7C0%7C1%7C638491229262880873%7CUnknown%7CTWFpbGZsb3d8eyJWIjoiMC4wLjAwMDAiLCJQIjoiV2luMzIiLCJBTiI6Ik1haWwiLCJXVCI6Mn0%3D%7C0%7C%7C%7C&sdata=jQ5rm5FC%2BIq087kDAaC7WGzYeKcFk9%2FGkWaU37wNU0I%3D&reserved=0)
2. (B)RUSET (2020). Tidskriften Alkohol & Narkotikas podd # 14 När, vem och med vilka berusar sig ungdomar. Intervju med Birgitta Ander. [https://podd.alkoholochnarkotika.se/](https://eur02.safelinks.protection.outlook.com/?url=https%3A%2F%2Fpodd.alkoholochnarkotika.se%2F&data=02%7C01%7CArne.Gerdner%40ju.se%7Cbbf9b6cc0abe4234e26208d7effbe8e8%7C7564bc8f37384b4dbd575a02ca6215fb%7C0%7C0%7C637241737529446052&sdata=TDBY5p3RTEKO%2B2sfT3tYDw80ndh210%2BZm2x0vXfLECI%3D&reserved=0)
3. Sabina Kapetanovic (2020). Hur skyddar man sin tonåring från droger och kriminalitet? Västpunkt: Från Högskolan Väst, Trollhättan. Länk: https://www.youtube.com/watch?v=frnwyYzyfbk
4. Arne Gerdner (2019): Presentation av LoRDIA. Förebygg.Nu. Första delen av följande inspelning. Länk: <https://www.youtube.com/watch?v=tEXNodFuCBg&t=1381s>
5. Birgitta Ander (2019). Ungdomars berusningsdrickande – vem, var och med vilka? LoRDIA presentationer från Förebygg.Nu. Andra delen av följande inspelning. Länk: <https://www.youtube.com/watch?v=tEXNodFuCBg&t=1381s>
6. Olof Aronsson och Arne Gerdner (2019). Social kontext och vänskap för ungdomar med invandrarbakgrund. Presentation på Förebygg.Nu, Tredje delen av följande inspelning. Länk: <https://www.youtube.com/watch?v=tEXNodFuCBg&t=1381s>
7. Karin Boson (2019). Tidigt berusningsdrickande och ungdomars riskbeteende. Förebygg.NU. Första delen av följande inspelning. Länk: <https://www.youtube.com/watch?v=6Ki5uyFnhcQ>
8. Russel Turner (2019). Ungdomars riskbeteenden vad gäller alkohol, droger och kriminalitet. Var finns risken? Presentation på Förebygg.NU. Andra delen av följande inspelning. Länk: <https://www.youtube.com/watch?v=6Ki5uyFnhcQ>
9. Sabina Kapetanovic (2019). Relationen mellan barn och föräldrar. Fokusspår: Att vara ung – jubileumssymposium om psykosociala faktorers påverkan på utvecklingen från barn till ung vuxen. Presentation på Förebygg.NU. Första (längre) delen av följande inspelning. Länk: https://www.youtube.com/watch?v=Ryrhz0z-AKI
10. Birgitta Lager och Maria Holmstrand (2019). Från en samverkande skola horisont. Exempel från Härryda. Presentation på Förebygg.NU. Andra (kortare) delen av följande inspelning. Länk: https://www.youtube.com/watch?v=Ryrhz0z-AKI
11. Russell Turner (2019). Ungdomar, alkohol, droger och kriminalitet. I serien ”Forskare förklarar”, CERA, Göteborgs universitet. Länk: [https://www.youtube.com/watch?v=64LrmhnO3ZU&list=PLiq9P8bruUJUPZks0BL5yDNafOJGCxfq-&index=14](https://eur02.safelinks.protection.outlook.com/?url=https%3A%2F%2Fwww.youtube.com%2Fwatch%3Fv%3D64LrmhnO3ZU%26list%3DPLiq9P8bruUJUPZks0BL5yDNafOJGCxfq-%26index%3D14&data=04%7C01%7CArne.Gerdner%40ju.se%7C7eea713ea17a44cfcf2308d8d59c511d%7C7564bc8f37384b4dbd575a02ca6215fb%7C0%7C1%7C637494214667064459%7CUnknown%7CTWFpbGZsb3d8eyJWIjoiMC4wLjAwMDAiLCJQIjoiV2luMzIiLCJBTiI6Ik1haWwiLCJXVCI6Mn0%3D%7C1000&sdata=HOTqmBG7d11SxU7la%2BVFe2dsk0tnZ%2Bdz23nuK%2BfZaJQ%3D&reserved=0)
12. Karin Boson (2019). Tidigt berusningsdrickande och ungdomars riskbeteende. Förebygg.NU. Länk: <https://www.youtube.com/watch?v=6Ki5uyFnhcQ>
13. Frida Lygnegård (2019). Vad påverkar ungdomars delaktighet? Finalist i Forskar Grand Prix. länk till UR Play: [https://urplay.se/program/215497-ur-samtiden-forskar-grand-prix-2019-vad-paverkar-ungdomars-delaktighet](https://eur02.safelinks.protection.outlook.com/?url=https%3A%2F%2Furplay.se%2Fprogram%2F215497-ur-samtiden-forskar-grand-prix-2019-vad-paverkar-ungdomars-delaktighet&data=02%7C01%7CArne.Gerdner%40ju.se%7Cd5e50b6a9b8648bcfd8208d7f0f73c8a%7C7564bc8f37384b4dbd575a02ca6215fb%7C0%7C0%7C637242816977383467&sdata=LGTMo0nJfTxWCxpaO1%2FC%2FyHYwyBA0TYIZSAEWUOJjIg%3D&reserved=0)

## Reports

1. Ylva Bjereld, Russell Turner & Lilly Augustine (Rapportmanus). Relationen mellan funktionsnedsättning, mobbning och mental hälsa över tid.
2. Arne Gerdner (2023). Code Book för LoRDIA:s kliniska intervjuer i våg 6 – dummyvariabler och instruktioner för prevalensberäkning. School of Health and Welfare, Department of Social Work.
3. Arne Gerdner (2021). Summarizing Report from Longitudinal Research on Development In Adolescence (LoRDIA). 25 February 2021. Jönköping University. School of Health and Welfare, Department of Social Work.
4. Arne Gerdner & Torbjörn Kalin (2021). Code Book för LoRDIA:s elevenkäter våg 1-5. LoRDIA-publikation från Jönköping University. School of Health and Welfare, Department of Social Work.
5. Arne Gerdner (2020). Code Book för LoRDIA:s lärarskattningar och skolregisterdata, Vågorna 1–4. LoRDIA-publikation från Jönköping University. School of Health and Welfare, Department of Social Work.
6. Arne Gerdner (2020). Code Book för LoRDIA:s föräldraenkäter Våg 1–2. LoRDIA-publikation från Jönköping University. School of Health and Welfare, Department of Social Work.
7. Arne Gerdner (2017). LoRDIAs Elevdata – svarspopulationer och bortfallsanalys. LoRDIA-publikation från Jönköping University. School of Health and Welfare, Department of Social Work.
8. Arne Gerdner (2017). Tonårsutveckling - Ett prospektivt longitudinellt forskningsprogram om ungdomars sociala nätverk, missbruk, psykiska hälsa och skolanpassning. Project number: 2012-25. Self-evaluation report to Formas, Vinnova, Vetenskapsrådet & FORTE.
9. Thorbjörn Ahlgren (2016). Powerpoint-rapport: Elevundersökning – X-Kommun. Kommunrapport nr 2 från LoRDIA. Jönköping University. School of Health and Welfare, Department of Social Work.
10. Thorbjörn Ahlgren (2016). Powerpoint-rapport: Elevundersökning – Y-Kommun. Kommunrapport nr 2 från LoRDIA. Jönköping University. School of Health and Welfare, Department of Social Work.
11. Thorbjörn Ahlgren (2016). Powerpoint-rapport: Elevundersökning – Z-Kommun. Kommunrapport nr 2 från LoRDIA. Jönköping University. School of Health and Welfare, Department of Social Work.
12. Thorbjörn Ahlgren (2016). Powerpoint-rapport: Elevundersökning – Q-Kommun. Kommunrapport nr 2 från LoRDIA. Jönköping University. School of Health and Welfare, Department of Social Work.
13. Thorbjörn Ahlgren (2014). Elevundersökning – X-Kommun. Rapport från LoRDIA. Jönköping University. School of Health and Welfare, Department of Social Work.
14. Thorbjörn Ahlgren (2014). Elevundersökning – Y-Kommun. Rapport från LoRDIA. Jönköping University. School of Health and Welfare, Department of Social Work.
15. Thorbjörn Ahlgren (2014). Elevundersökning – Z-Kommun. Rapport från LoRDIA. Jönköping University. School of Health and Welfare, Department of Social Work.
16. Thorbjörn Ahlgren (2014). Elevundersökning – Q-Kommun. Rapport från LoRDIA. Jönköping University. School of Health and Welfare, Department of Social Work.
