# Supplementary material for: The Swedish longitudinal Gothenland Millennium Cohort for studying wellbeing from early adolescence through adulthood
Source: BMJ Open. 2025 Dec 10;15(12):e100327. doi: 10.1136/bmjopen-2025-100327 (PMC12699670; doi:10.1136/bmjopen-2025-100327)
Supplement: Supplementary file 2 [file bmjopen-2025-100327.reviewer_comments.pdf]

PEER REVIEW HISTORY

BMJ Open publishes all reviews undertaken for accepted manuscripts. Reviewers are asked to complete a checklist review form and are provided with free text boxes to elaborate on their assessment. These free text comments are reproduced below.

ARTICLE DETAILS

Title (Provisional)

Cohort Profile: The Swedish longitudinal Gothenland Millennium Cohort for studying wellbeing from early adolescence through adulthood

Authors

Olsson, Tina M.; Kalin, Torbjörn; Kapetanovic, Sabina; Turner, Russell; Gerdner, Arne

---

VERSION 1 - REVIEW

Reviewer 1

Name Harding, Seeromanie

Affiliation King\'s College London Division of Diabetes and Nutritional Sciences, Diabetes & Nutritional Sciences Division

Date 29-Apr-2025

COI None

I appreciated the opportunity to learn about the Swedish longitudinal Gothenburg Millennium Cohort. This cohort represents a valuable and ambitious initiative for examining wellbeing across the transition from adolescence into adulthood. Its design is particularly impressive, featuring a rich array of multi-informant data collected over time from adolescents, parents, and teachers, in addition to linked administrative records. The cohort has already contributed to numerous peer-reviewed publications, conference presentations, and doctoral theses speaks to its importance and utility within the research community.

As this is a cohort profile article, I have general comments for the authors.

First, given the substantial attrition across study waves—particularly the 50.4% participation rate in Wave 5—it is important for the authors to provide a more detailed analysis of the representativeness of the remaining participants. In particular, examining whether those who dropped out differ systematically from those who continued, based on key sociodemographic characteristics collected in earlier waves (e.g., gender, socioeconomic status, immigration background, parental education), would be crucial for assessing the extent of attrition bias and the generalisability of findings.

Second, the relatively low parental response rates in Waves 1 (32%) and 2 (41%) raise concerns about potential response bias in analyses that incorporate parental data. The study would be strengthened if the authors explored whether there were systematic demographic differences between adolescents whose parents responded and those whose parents did not.

In light of these considerations, the authors should reflect on whether constructing baseline survey weights and/or attrition weights—and applying them in future analyses—would be appropriate to address potential biases arising from non-random participation and dropout. The application of such weights could enhance the representativeness of the analytic sample and improve the robustness of longitudinal findings. If weighting procedures were not applied, it would be helpful for the authors to provide a rationale for this decision. Addressing these issues would further strengthen the internal validity of the study and enhance the interpretability of its longitudinal results.

Reviewer **2**

Name **Kvernmo, Siv**

Affiliation **UiT The Arctic University of Norway, Department of Clinical Medicine**

Date **12-Sep-2025**

COI **None**

I thank you for the opportunity to review this interesting and valuable paper. This paper describes the Gothenland Millennium Cohort research program. The multi-disciplinary program covers a broad spectrum of topics in adolescents´ lives and their pathways from early adolescence into adulthood. An impressive work is described. By covering both the aims, the design and methodology, a summary of key findings, strengths and limitations but also the impact of the study, the reader gets detailed information of the research process. The purpose of the present paper is to stimulate collaboration and use of the data from the program.

My general opinion is that this is an excellent written paper, which is useful for researchers planning a longitudinal prospective study of mental health and well-being in children and adolescents but also for new knowledge of adolescent health

In the . I only have minor recommendations. First, in the abstract I think there is a need to briefly describe the purpose of this paper. Also in the Introduction part I think that the purpose of the paper should be mentioned earlier in the section.

Although several measures is named in the paper, I get an impression that more measures are used than those mentioned. Therefore, I will suggest that a table or a supplementary table of all most important measures used ,could be helpful for the reader.

The section "Findings to date" is particularly interesting and impressive, as it covers a broad range of research areas. However, I missed information about the use of social media and its impact by the adolescents. As this is an important factor to mental health and well-being nowadays, it could be mentioned if this was measured or not.

VERSION 1 - AUTHOR RESPONSE

Reviewer 1 comments

1. Given the substantial attrition across study waves—particularly the 50.4% participation rate in Wave 5—it is important for the authors to provide a more detailed analysis of the representativeness of the remaining participants. In particular, examining whether those who dropped out differ systematically from those who continued, based on key sociodemographic characteristics collected in earlier waves (e.g., gender, socioeconomic status, immigration background, parental education), would be crucial for assessing the extent of attrition bias and the generalisability of findings.

   Thank you for this important comment. We have now included an analysis of baseline characteristics for those that dropped out and those that remained in the study at Wave 5. This information in included in the section Attrition.

2. The relatively low parental response rates in Waves 1 (32%) and 2 (41%) raise concerns about potential response bias in analyses that incorporate parental data. The study would be strengthened if the authors explored whether there were systematic demographic differences between adolescents whose parents responded and those whose parents did not.

   Thank you for this important comment. We have now included an analysis of baseline characteristics for those that dropped out and those that remained in the study at Wave 5. This information in included in the section Attrition.

3. In light of these considerations, the authors should reflect on whether constructing baseline survey weights and/or attrition weights—and applying them in future analyses—would be appropriate to address potential biases arising from non-random participation and dropout. The application of such weights could enhance the representativeness of the analytic sample and improve the robustness of longitudinal findings. If weighting procedures were not applied, it would be helpful for the authors to provide a rationale for this decision. Addressing these issues would further strengthen the internal validity of the study and enhance the interpretability of its longitudinal results.

Thank you, we have now included a reflection of this issue in the study limitations section.

Reviewer 2 comments

4. In the abstract I think there is a need to briefly describe the purpose of this paper. Also, in the Introduction part I think that the purpose of the paper should be mentioned earlier in the section.

   Thank you. We have added the purpose of this paper to the abstract.

5. Although several measures is named in the paper, I get an impression that more measures are used than those mentioned. Therefore, I will suggest that a table or a supplementary table of all most important measures used ,could be helpful for the reader.

   Thank you. Our (adolescent self-report) data includes over 400 variables, as such we chose therefore to present measures by overarching theme as opposed to detailing each variable. We have added text which states that interested parties are welcome to contact with us for details on the included variables. We hope that this is sufficient.

6. The section "Findings to date" is particularly interesting and impressive, as it covers a broad range of research areas. However, I missed information about the use of social media and its impact by the adolescents. As this is an important factor to mental health and well-being nowadays, it could be mentioned if this was measured or not.
